# High miR156 Expression Is Required for Auxin-Induced Adventitious Root Formation via *MxSPL26* Independent of *PINs* and *ARFs* in *Malus xiaojinensis*

**DOI:** 10.3389/fpls.2017.01059

**Published:** 2017-06-19

**Authors:** Xiaozhao Xu, Xu Li, Xingwang Hu, Ting Wu, Yi Wang, Xuefeng Xu, Xinzhong Zhang, Zhenhai Han

**Affiliations:** Institute for Horticultural Plants, College of Horticulture, China Agricultural UniversityBeijing, China

**Keywords:** adventitious rooting, auxin, leafy cutting, *Malus xiaojinensis*, miR156

## Abstract

Adventitious root formation is essential for the vegetative propagation of perennial woody plants. During the juvenile-to-adult phase change mediated by the microRNA156 (miR156), the adventitious rooting ability decreases dramatically in many species, including apple rootstocks. However, the mechanism underlying how miR156 affects adventitious root formation is unclear. In the present study, we showed that in the presence of the synthetic auxin indole-3-butyric acid (IBA), semi-lignified leafy cuttings from juvenile phase (Mx-J) and rejuvenated (Mx-R) *Malus xiaojinensis* trees exhibited significantly higher expression of miR156, *PIN-FORMED1* (*PIN1*), *PIN10*, and *rootless concerning crown and seminal roots*-like (*RTCS*-like) genes, thus resulting in higher adventitious rooting ability than those from adult phase (Mx-A) trees. However, the expression of *SQUAMOSA-PROMOTER BINDING PROTEIN-LIKE26* (*SPL26*) and some *auxin response factor* (*ARF*) gene family members were substantially higher in Mx-A than in Mx-R cuttings. The expression of *NbRTCS*-like but not *NbPINs* and *NbARFs* varied with miR156 expression in tobacco (*Nicotiana benthamiana*) plants transformed with *35S:MdMIR156a6* or *35S:MIM156* constructs. Overexpressing the miR156-resistant *MxrSPL* genes in tobacco confirmed the involvement of *MxSPL20, MxSPL21*&*22*, and *MxSPL26* in adventitious root formation. Together, high expression of miR156 was necessary for auxin-induced adventitious root formation via *MxSPL26*, but independent of *MxPINs* and *MxARFs* expression in *M. xiaojinensis* leafy cuttings.

## Introduction

Adventitious rooting is a cornerstone of proliferation for most fruit and forest species that are vegetatively propagated from elite genotypes. In the apple rootstocks, *Sequoia sempervirens* and *Pinus radiata* for example, propagation via both hard wood and leafy cuttings are constrained by adventitious rooting recalcitrance in the reproductively competent donor trees (Chen et al., 1981; Huang et al., 1992; Sanchez et al., 2007). To date, several techniques have been developed to improve rooting ability in some woody perennials, such as cycles of *in vitro* culture of apple rootstocks, or the repeated grafting of adult scions onto juvenile rootstocks in English ivy (*Hedera helix* L.) and chestnut (*Castanea sativa*; Giovannelli and Giannini, 2000; Xiao et al., 2014). The regulation of adventitious rooting is, however, relatively unexplored.

Adventitious root formation depends on multiple factors, such as genetic background, developmental stage, hormones, and other internal and external cues (Geiss et al., 2009; da Costa et al., 2013). In most tree species, the ability to form adventitious roots decreases during the transition from juvenile to adult development phases. In *S. sempervirens*, the rooting rate of cuttings from juvenile trees was up to 100% compared to 30% in cuttings from adult trees (Huang et al., 1992). Similarly, the rooting rate of cuttings from 3-year-old juvenile plants was significantly higher (88%) than from 36-year-old adult plants (17%) of Carolina Buckthorn (*Rhamnus caroliniana* Walt.; Graves, 2002). In our previous study, rooting rates of cuttings from juvenile, juvenile-like, and rejuvenated donor plants were significantly higher (77%) than those of cuttings from adult trees (11%) in *Malus xiaojinensis* (Xiao et al., 2014).

Numerous genes are differentially expressed between juvenile and adult cuttings prior to root induction. In loblolly pine (*Pinus taeda* L.), *5NG4*, a nodulin-like gene, is highly and specifically induced by auxin in juvenile shoots prior to adventitious root formation, but is then substantially down-regulated in physiologically mature shoots that are adventitious rooting incompetent (Busov et al., 2004). A gene encoding a nitrate reductase involved in nitric oxide production in *Eucalyptus grandis* is up-regulated in juvenile cuttings as compared to that in mature cuttings, which might lead to increased ability to produce nitric oxide and form adventitious roots (Abu-Abied et al., 2012). Transcriptome data showed that the expression of *E. grandis* homologs of *Peroxidase 72, PIN3*, and *Aux/IAA 19* (*IAA19*) were higher at a certain time point in auxin treated juvenile cuttings compared to mature ones (Abu-Abied et al., 2014); however, the insight effect of juvenility on adventitious root formation is not clear.

The juvenile to adult phase change is initiated by a decrease in the expression of the miR156. In *Arabidopsis*, maize (*Zea mays*) and various woody plants including *Acacia confusa, Acacia colei, E. globulus, H. helix, Quercus acutissima*, and *Populus* × *Canadensis*, miR156 is highly abundant in seedlings and decreases in adult plants (Wu and Poethig, 2006; Chuck et al., 2007; Wang et al., 2011). In the *Congrass1* maize mutant that overexpresses miR156, prop roots are produced at all nodes in the plant, while these roots only grow from shoot-born meristem at the juvenile nodes in wild-type plants (Chuck et al., 2007). Similarly, tomato and tobacco plants overexpressing miR156 exhibit dense aerial roots on their stems, while none appear on the stems of wild type plants (Zhang et al., 2011; Feng et al., 2016). In contrast, *Arabidopsis thaliana* plants transformed with *35S:MIM156* produce significantly fewer adventitious roots from the base of the hypocotyl than wild-type plants (Xu et al., 2016).

MiR156 acts by repressing the expression of a group of *SQUAMOSA-PROMOTER BINDING PROTEIN-LIKE* (*SPL*) genes. Elevated levels of miR156 promote adventitious root formation in maize, tomato, and tobacco, indicating that SPL proteins inhibit adventitious root formation (Chuck et al., 2007; Zhang et al., 2011; Feng et al., 2016). *EgSPL2* and *EgSPL5* are up-regulated in mature cuttings compared to juvenile cuttings of *E. grandis*, and the juvenile cuttings exhibited a higher adventitious rooting percentage (Abu-Abied et al., 2012). These results suggest that miR156 and miR156 putative targeted *MxSPL* genes may play important roles in adventitious root formation during the juvenile to adult phase change. Twenty-seven *SPL* gene family members were identified in the apple genome and 13 were predicted to be targets of miR156 using degradome sequencing (Li et al., 2013; Xing et al., 2014). However, which *SPL* gene members are involved in adventitious root formation is unknown.

Auxin is an effective inducer of adventitious root formation; the dosage and gradient of, and response to, auxin are all important for plant root growth. Synthetic auxins like indole-3-butyric acid (IBA) have been used for almost 80 years to induce adventitious rooting (Zimmerman and Wil-Coxon, 1935). Without IBA treatment, leafy cuttings from juvenile *M. xiaojinensis* do not form adventitious roots (Xiao et al., 2014). The gradient of auxin controls adventitious root formation; in detached *Arabidopsis* leaves, an auxin gradient is required in the procambium cells during adventitious root formation (Liu J. C. et al., 2014). Auxin efflux carriers, such as PIN-FORMED (PIN) proteins, establish auxin concentration gradients (Yang and Murphy, 2009; Adamowski and Friml, 2015). Transgenic data suggests that *OsPIN1* plays an important role in auxin-dependent adventitious root emergence in rice (Xu et al., 2005). Furthermore, *PtoPIN1c* is induced and maintained at a 20-fold level during the adventitious root initiation phase in *Populus* (Liu B. B. et al., 2014). In *Arabidopsis*, the *pin1-1* mutant formed 40% fewer adventitious roots than wild type (Sukumar et al., 2013). All of these results demonstrate that regeneration of adventitious roots requires polar auxin transport.

Beyond auxin gradients and dosage, genes that participated in auxin signaling pathways are also involved in adventitious root formation. The transgenic *Arabidopsis* line overexpressing the *auxin response factor 17* (*ARF17*) gene develops fewer adventitious roots, while plants overexpressing the *ARF6* or *ARF8* develop more adventitious roots than wild-type plants (Sorin et al., 2005; Gutierrez et al., 2009). It was previously reported that to promote adventitious root formation, ARF proteins directly regulate *LATERAL ORGAN BOUNDARIES DOMAIN PROTEIN* (*LBD*) genes by binding to their promoter sequences (Okushima et al., 2007; Majer et al., 2012). *Crown rootless 1* (*Crl1*) encodes a member of the *LBD* genes in rice (*Oryza sativa*), which positively regulate adventitious root formation, and its expression is directly regulated when an ARF binds to the 5′ flanking sequences of *Crl1* (Inukai et al., 2005). In maize, *rootless concerning crown and seminal root* (*Rtcs*) encodes a LBD protein that positively regulates adventitious root growth, and its expression is regulated by ZmARF34, which binds to an auxin response element in the promoter region of *Rtcs* (Majer et al., 2012). However, during the adventitious root formation in recalcitrant woody plants, whether and how miR156 interacts with auxin remains unknown.

To evaluate how juvenility mediates adventitious rooting in woody plants and using apple rootstock *M. xiaojinensis* as an example, we analyzed the transcript level of miR156, miR156 putative target *SPL* gene expression, and *MxPIN* and *MxARF* gene family members during the adventitious rooting process. Then, the function of miR156 in adventitious root formation was validated by generating transgenic tobacco lines. The results elucidate the role of miR156 in adventitious root formation and will be useful in horticultural and forestry industries.

## Materials and methods

### Plant material

*M. xiaojinensis* (Mx) was used in these experiments because of its high apomictic rate to ensure stable and robust juvenile materials (Li et al., 2004). The apomictic origin of donor trees used for leafy cutting collection has been confirmed by simple sequence repeat (SSR) and single nucleotide polymorphism (SNP) markers (Wang et al., 2012). Cuttings were excised from basal suckers (juvenile phase, Mx-J), shoots from the canopy of reproductively mature trees (adult phase, Mx-A), and rejuvenated plants via *in vitro* apical meristem culture (rejuvenated or juvenile like, Mx-R; Xiao et al., 2014).

Semi-lignified leafy cuttings (8–10 cm in length) were excised from actively growing shoots of donor plants, dipped 1 cm in depth into a solution containing 3000 mg/L indole butyric acid (IBA) (V900325, Sigma-Aldrich, St. Louis, MO, USA) for 1 min, and then the cuttings were plugged into 50 cell trays containing fine sand as a rooting medium. The cuttings were incubated in a solar greenhouse under 90–95% relative humidity (Xiao et al., 2014). Cuttings treated with an IBA-free medium solution were used as controls. The experimental errors were managed by using a complete randomized design for three biological replicates, each including at least 50 leafy cuttings. Rooting parameters were scored at 35 d after application (Xiao et al., 2014).

### Plant expression vector construction and transgenic tobacco generation

Nine miR156 precursor genes were previously identified in apple genome (Ma et al., 2014). *MdMIR156a6* (Genome location: MDC018927.245), which relative transcript level was higher than the other members, was chosen for overexpression study (Supplementary Figure 1). The apple *MdMIR156a6* DNA fragment was amplified from *Malus domestica* genomic DNA. Artificial target mimics were generated by modifying the sequence of the *AtIPS1* gene to knock-down miR156 expression (Franco-Zorrilla et al., 2007). The PCR products were sequenced by BGI (Shenzhen, China). All constructs were cloned behind the constitutive CaMV 35S promoter in the pBI121 vector, and then introduced into *Nicotiana benthamiana* by *Agrobacterium tumefaciens*-mediated transformation (Horsch et al., 1985).

*MxSPLs* coding sequences were amplified from *M. xiaojinensis* cDNA. The miR156-resistant *SPLs* (*rSPLs*) were made by two rounds of mutagenic PCR using KOD-Plus Nero DNA polymerase (KOD-401, TOYOBO LIFE SCIENCE, Japan), and sequencing confirmed the mutations by BGI (Shenzhen, China). The *rSPLs* variants were driven by the CaMV 35S promoter in the pBI121 vector. These constructs were introduced into *35S:MdMIR156a6* transgenic tobacco leaves by *A. tumefaciens*-mediated transformation. The infected leaves were selected on MS medium supplemented with 100 mg/L kanamycin and 300 mg/L cefotaxime sodium to generate *rSPL* and miR156 co-overexpressing transgenic lines. All primers are listed in Supplementary Table 1. Transgenic tobacco plants were proliferated by subculture on hormone-free MS medium. Rooting rate and the number of adventitious roots were recorded at 5, 7, 9, 11, and 13 d after subculture. Plants were incubated at 25°C in long-day light conditions.

### Histological analysis

Mx-J, Mx-A, and Mx-R cutting samples were collected at 0, 14, and 28 d after IBA treatment; cuttings from *35S:MdMIR156a6* and *35S:MIM156* transgenic tobacco lines were excised at 0, 3, and 6 d after subculture on hormone-free MS medium. All samples were collected from three biological replicates and *n* = 10 in each replicate. A one-centimeter section from the bottom of each cutting was excised and fixed in a 5:50:5 (v/v/v) formaldehyde/ethanol/acetic acid (FAA) solution overnight at room temperature. Cuttings were dehydrated in an ethanol series (70, 85, 95, and 100%), infiltrated with xylene, and embedded in paraffin. Then, 15 μm-thick transverse sections were cut with a rotatory microtome (KD-2258, KEDEE, China) and stained with toluidine blue (Rigal et al., 2012).

### Gene expression analysis

For relative gene expression assays with *M. xiaojinensis*, stem bark from 0.5 to 1 cm basal sections of 20 cuttings were frozen in liquid nitrogen at 0, 6, 12, 24, 72, 120, and 168 h after IBA treatment. For gene expression profile analysis in tobacco, stems from 0.5 to 1 cm basal sections of 20 cuttings were sampled at 0, 3, 6, 12, 24, 48, and 72 h after subculture on hormone-free MS medium. Total RNA was isolated from ~500 mg of frozen tissue using a modified cetyltrimethylammonium bromide (CTAB) method (Gasic et al., 2004). The RNA was digested by DNaseI (2313A, Takara, Dalian, China) and reverse-transcribed using oligo-dT18 primers and reverse transcriptase according to the manufacturer's instructions (2641A, Takara, Dalian, China). Semi-quantitative RT-PCR results were quantified by using ImageJ 1.47v (Wayne Rasband, National Institutes of Health, USA) according to the commands in “gels submenu” to analyze one-dimensional electrophoretic gels (https://imagej.nih.gov/ij/docs/menus/analyze.html#gels). Quantitative RT-PCR were performed using SYBR green reagents (RR820A, Takara, Dalian, China) in an Applied Biosystems 7500 real-time PCR system. *M. xiaojinensis EF1*α or *N. benthamiana EF1*α was used as the expression control for these experiments. The relative expression level was calculated according to the 2^−ΔΔCT^ method (Livak and Schmittgen, 2001). Three independent biological replicates and technical replicates were performed.

MicroRNA was extracted by using the RNAiso for Small RNA kit (9753Q, Takara, Dalian, China) according to the manufacturer's instruction. MiR156 expression level was analyzed by qRT-PCR as described previously (Xiao et al., 2014). The apple *PIN* and *ARF* family genes were previously identified (Devoghalaere et al., 2012; Zhang H. et al., 2015). The apple and *N. benthamiana RTCS-like* gene were selected by using a BLASTP search of known Maize *RTCS* (GenBank accession number EF051732) gene against the Apple Genome Database (https://www.rosaceae.org/) and Tobacco Genome Database (https://www.solgenomics.net/), respectively. *N. benthamiana PIN* and *ARF* genes were selected by using a BLASTP search of known *Arabidopsis* auxin-related genes against the Tobacco Genome Database (https://www.solgenomics.net/). All primers used for qRT-PCR are listed in Supplementary Tables 2,3.

### Statistical analysis

Statistical analysis was performed using the Statistical Product and Service Solutions (SPSS) software (IBM Co., Armonk, USA). All experimental data were tested by Student's *t-*test or Duncan's multiple-range test.

## Results

### Adventitious rooting ability of Mx-A, Mx-J, and Mx-R leafy cuttings

**Figure 1:**
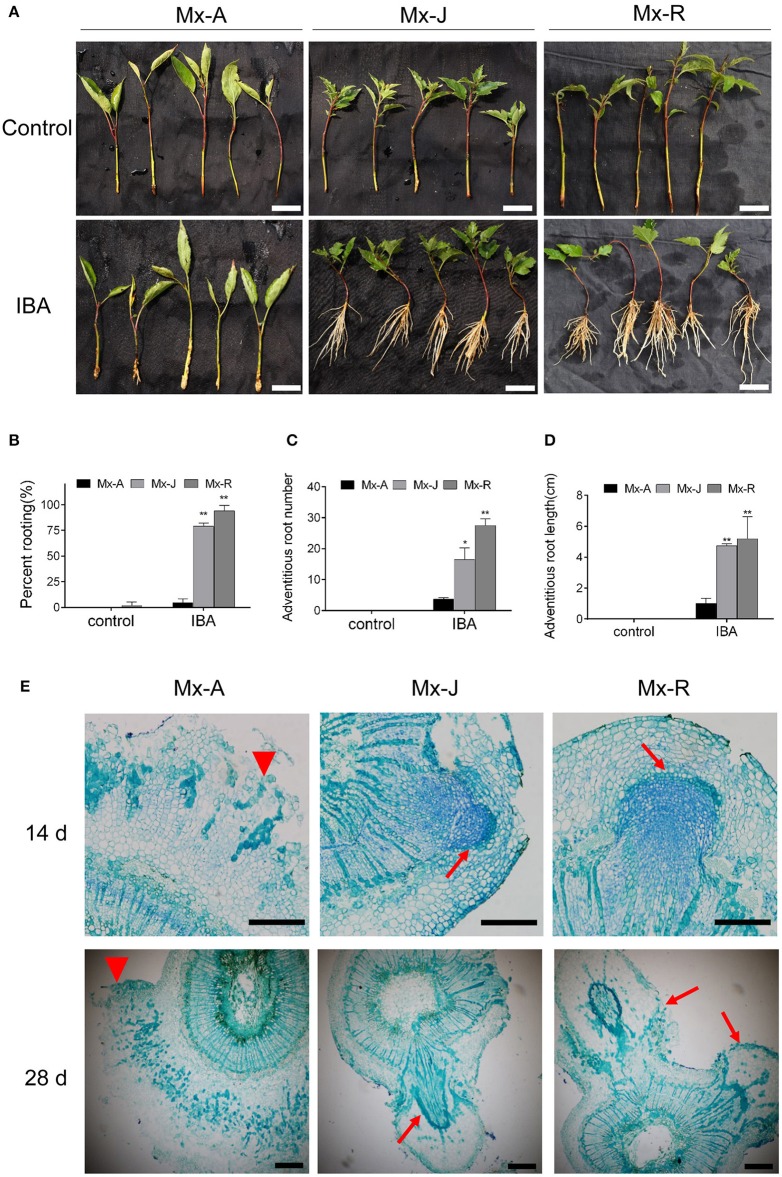
Adventitious rooting ability of adult phase (Mx-A), juvenile phase (Mx-J) and rejuvenated (Mx-R) leafy cuttings of *Malus xiaojinensis*. **(A)** Morphological features, **(B)** rooting percent, **(C)** adventitious root number, and **(D)** adventitious root length of Mx-A, Mx-J, and Mx-R cuttings at 35 d of IBA or control treatments. Scale bars = 20 mm in **(A)**. **(B–D)** Bars show *SD* from three biological replicates. *n* = 50 individuals in each replicate. Asterisks indicate significant difference from the Mx-A (Student's *t*-test, ^**^P < 0.01, ^*^P < 0.05). **(E)** Histological features of Mx-A, Mx-J, and Mx-R cuttings during adventitious root formation at 14 days (upper) and 28 days (bottom) after IBA treatment. Toluidine Blue-stained cross section of the stem of Mx-A, Mx-J, and Mx-R at 14 and 28 days after IBA treatment. Arrows indicate emerging adventitious root initials. Scale bars = 200 μm in **(E)**.

Juvenile cuttings (Mx-J) exhibited a highly adventitious rooting ability in *M. xiaojinensis* (Figure 1A). Leafy Mx-J cuttings exhibited a 78.63% rooting percentage at day 35 after IBA treatment (Figure 1B). In contrast, the rooting percentage of adult cuttings (Mx-A) was significantly lower (4.38%) than that of Mx-J. The adventitious root number per cutting was also lower in Mx-A cuttings than in Mx-J cuttings (Figure 1C). Similarly, the adventitious root length in Mx-J was significantly longer than that in Mx-A cuttings. Mx-R cuttings exhibited significantly enhanced adventitious rooting percentage and adventitious root number than Mx-A or Mx-J cuttings (Figure 1).

To determine whether adult leafy cuttings have defects in the initiation of adventitious root formation primordia, cuttings were processed for histological analysis after IBA treatment. There were no obvious differences in anatomical structure between juvenile and adult softwood cuttings before IBA treatment (Supplementary Figure 2). At 14 d after IBA treatment, root primordia appeared in Mx-J and Mx-R but not Mx-A cuttings. At 28 d, root primordia penetrated the stem cortex and epidermis, and projected well beyond the stem surface in Mx-J and Mx-R (Figure 1E). In contrast, although meristematic cambium cells were observed between the phloem and xylem layers, except for callus, there was almost no differentiated root primordia detected in Mx-A cuttings even after 28 d of auxin treatment (Figure 1E).

### The relationship between miR156 expression and adventitious root formation

**Figure 2:**
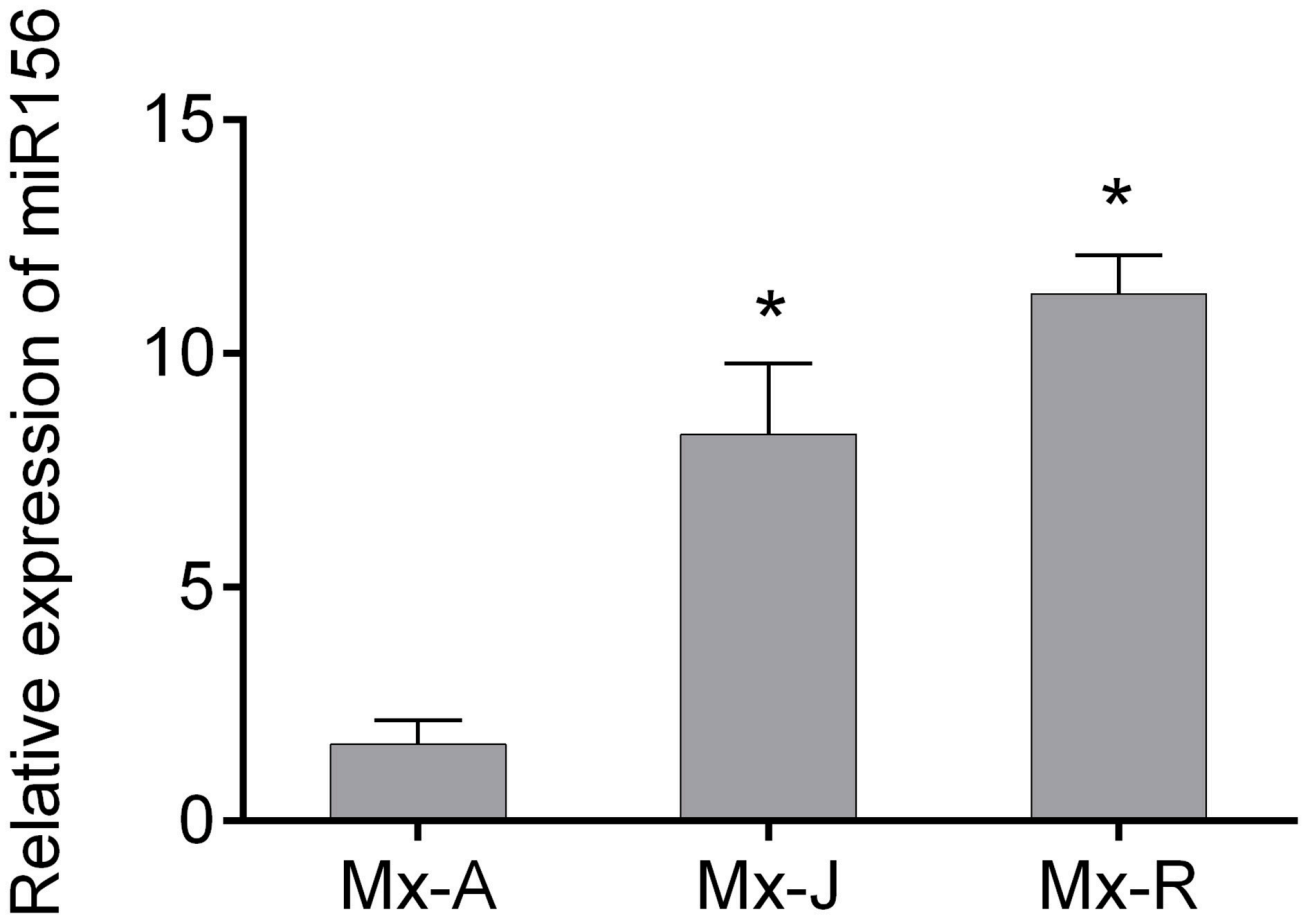
Expression level of miR156 in adult phase (Mx-A), juvenile phase (Mx-J), and rejuvenated (Mx-R) leafy cuttings of *Malus xiaojinensis*. MicroRNA was extracted from Mx-A, Mx-J, and Mx-R cuttings before IBA treatment. Relative expression was measured with quantitative real time PCR and normalized to that *5S rRNA*. Asterisks indicate statistical significance (^*^P < 0.05) in comparison with Mx-A. Bars show *SD* from three biological replicates.

**Figure 3:**
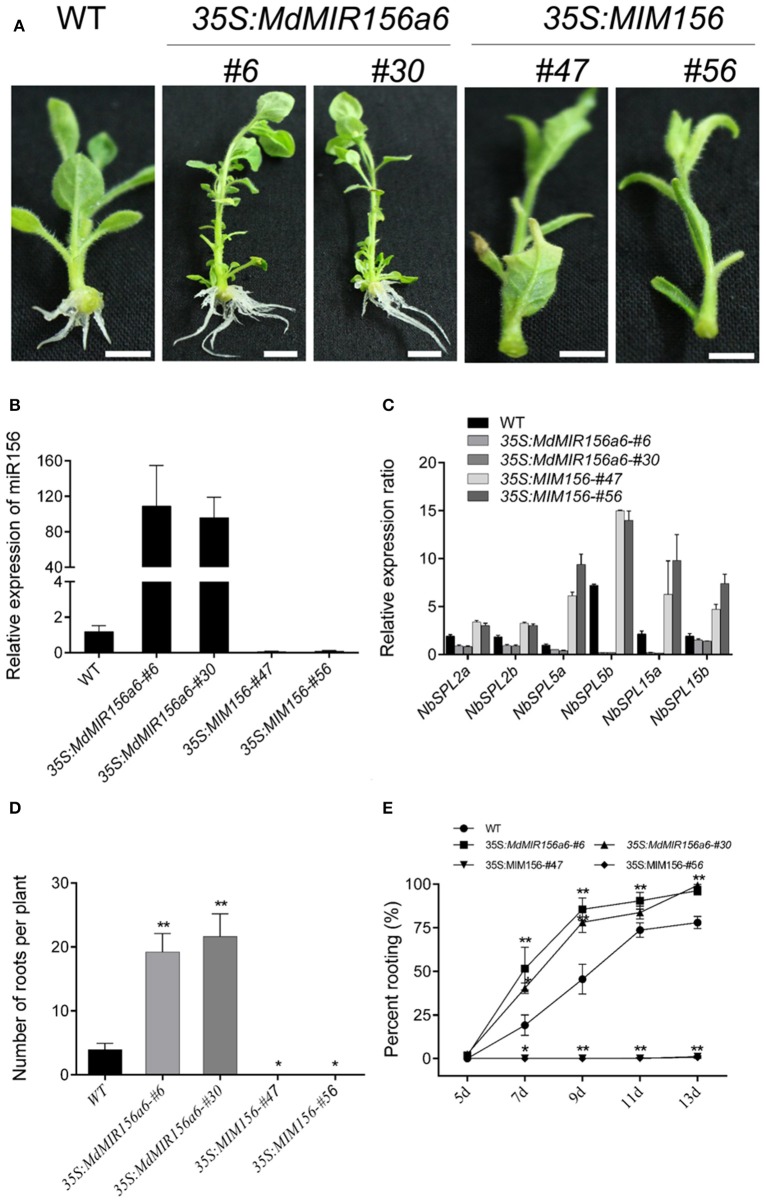
MiR156 affects adventitious rooting capacity in transgenic tobacco plants. **(A)** Phenotypes of 13-day-old plants (wild-type, *35S:MdMIR156a6*, and *35S:MIM156*) grown on hormone-free MS medium. Scale bars = 20 mm. **(B)** Relative expression level of miR156 in transgenic tobacco lines. Bars show *SD* from three biological replicates. **(C)** Relative expression levels of *NbSPLs* in WT, *35S:MdMIR156a6*, and *35S:MIM156* plants in stems. **(D,E)** Rooting ability of tobacco stem cuttings. **(D)** Adventitious root number per cutting and **(E)** percent rooting. Adventitious root number was counted after 13 days of growth on MS medium. Bars show *SD* from three biological replicates; *n* = 10 in individuals per replicate. Asterisks indicate significant differences from the WT (Student's *t*-test, ^**^P < 0.01, ^*^P < 0.05).

Consistent with the rooting ability, the expression level of miR156 in Mx-J and Mx-R cuttings were significantly higher than that in Mx-A cuttings (Figure 2). To validate if miR156 regulates adventitious root formation, we generated transgenic tobacco plants expressing *35S:MdMIR156a6* or *35S:MIM156* to enhance or inhibit miR156 activity, respectively (Figure 3A). Mature miR156 expression levels were significantly up-regulated in *35S:MdMIR156a6* plants and significantly reduced in *35S:MIM156* plants (Figure 3B). Similarly, the expression levels of some miR156 target genes, including *NbSPL2a, NbSPL2b, NbSPL5a, NbSPL5b, NbSPL15a*, and *NbSPL15b* were down-regulated at least 2-fold in *35S:MdMIR156a6* stems, but at least 5-fold up-regulated in *35S:MIM156* stems (Figure 3C). *35S:MdMIR156a6* plants developed almost 20 adventitious roots per cutting, which was 4-fold more than the wild type after 13 days of culture on MS medium. Almost no adventitious roots were observed in *35S:MIM156* cuttings (Figure 3D). In addition to the difference in adventitious root number, the rate of adventitious root development in *35S:MdMIR156a6* plants was significantly faster than that measured in the wild-type plants (Figure 3E).

**Figure 4:**
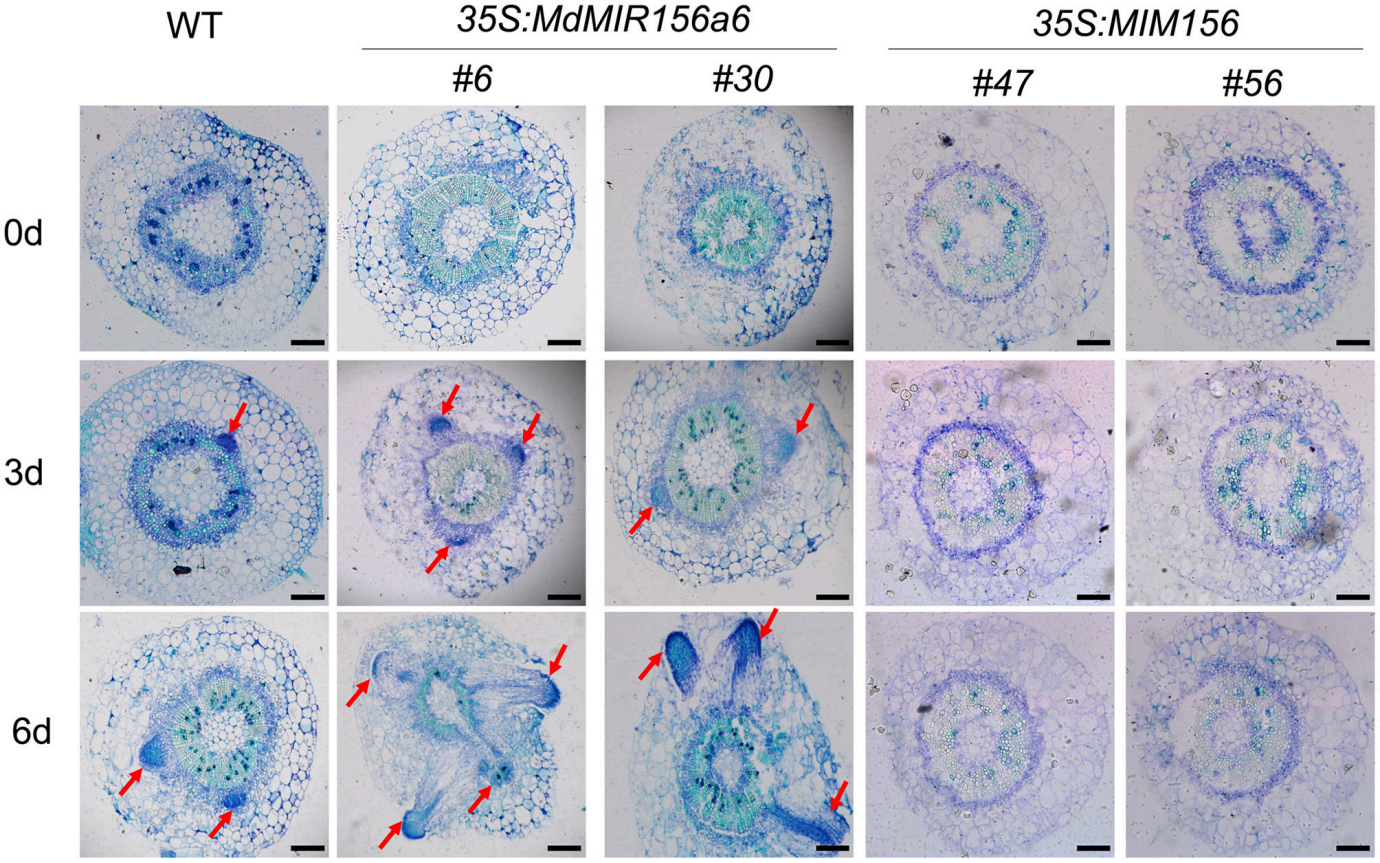
Histological features of WT, *35S:MdMIR156a6*, and *35S:MIM156* tobacco stems during adventitious root formation. Toluidine Blue-stained cross-section of the stem of WT, *35S:MdMIR156a6*, and *35S:MIM156* at 0, 3, and 6 d after subculture on MS medium. Arrows indicate initial emerging adventitious roots and adventitious root primordia. Scale bars = 200 μm.

To check whether miR156 affects the initiation of adventitious root primordia, serial cross sections of the stems of wild-type, *35S:MdMIR156a6* expressing transgenic lines, and *35S:MIM156* expressing transgenic tobacco plants were stained with toluidine blue. When compared with the wild-type, the initiation of adventitious root primordia was accelerated and well-developed in *35S:MdMIR156a6* stem cuttings only 3 days after subculture on MS (Figure 4). By contrast, no adventitious root primordia were observed in *35S:MIM156* stem cuttings throughout the experiment (Figure 4).

### Interaction between miR156 and auxin during adventitious root formation

**Figure 5:**
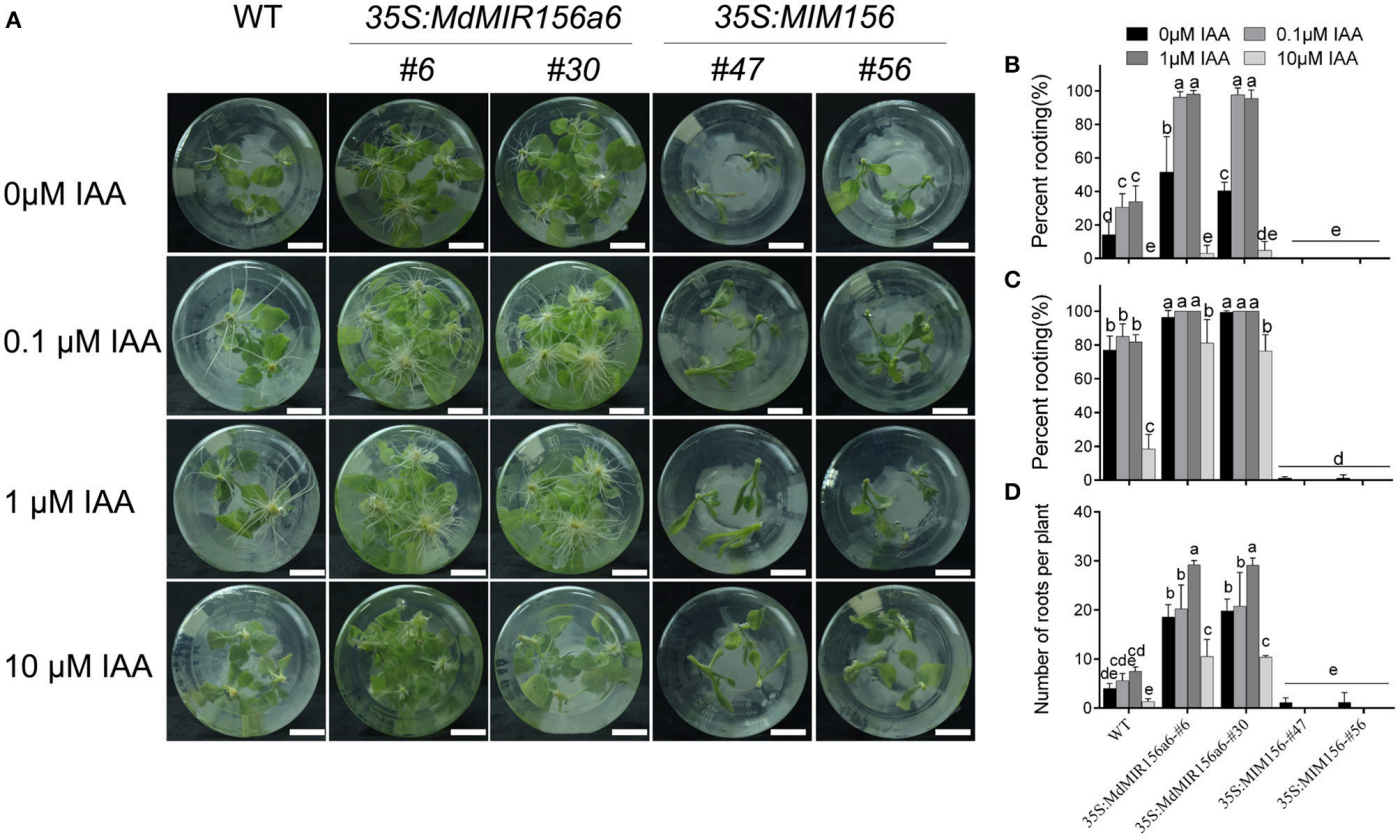
Effects of IAA on adventitious root formation of *35S:MdMIR156a6* and *35S:MIM156* tobacco plants. **(A)** Phenotypes of 13-day-old plants (WT, *35S:MdMIR156a6*, and *35S:MIM156*) grown on MS medium with or without IAA treatment. Scale bars = 15 mm. **(B,C)** Percent rooting of tobacco stem cuttings grown on MS medium for 7 and 14 d, respectively. **(D)** Adventitious root number per cutting. Adventitious root number was counted after 14 d on MS medium. Bars show *SD* with three biological replicates; *n* = 5 cuttings in each replicate. The statistical analysis was performed by Duncan's multiple range test at level *p* ≤ 0.05. The same lowercase letter indicate no significant differences.

To determine whether miR156 regulates adventitious root formation through modulating endogenous auxin levels or auxin polar transport, we examined adventitious rooting capacity in wild-type, *35S:MdMIR156a6*, and *35S:MIM156* stem cuttings grown on MS medium supplemented with different concentrations of indole-3-acetic acid (IAA) or 1-N-naphthylphthalamic acid (NPA). The percent rooting was significantly increased in wild-type and *35S:MdMIR156a6* stem cuttings when cultured in the presence of 0.1 or 1 μM IAA (Figures 5A,B). The 10 μM IAA treatment delayed adventitious root formation in both wild-type and *35S:MdMIR156a6* plants; however, *35S:MdMIR156a6* plants exhibited a significantly higher adventitious rooting percent and adventitious root number than wild-type. In addition, with IAA application, the adventitious root number was clearly increased in both wild-type and *35S:MdMIR156a6* plants (Figures 5A,C) but IAA treatment did not rescue the adventitious rooting capacity defect in *35S:MIM156* plants (Figure 5).

**Figure 6:**
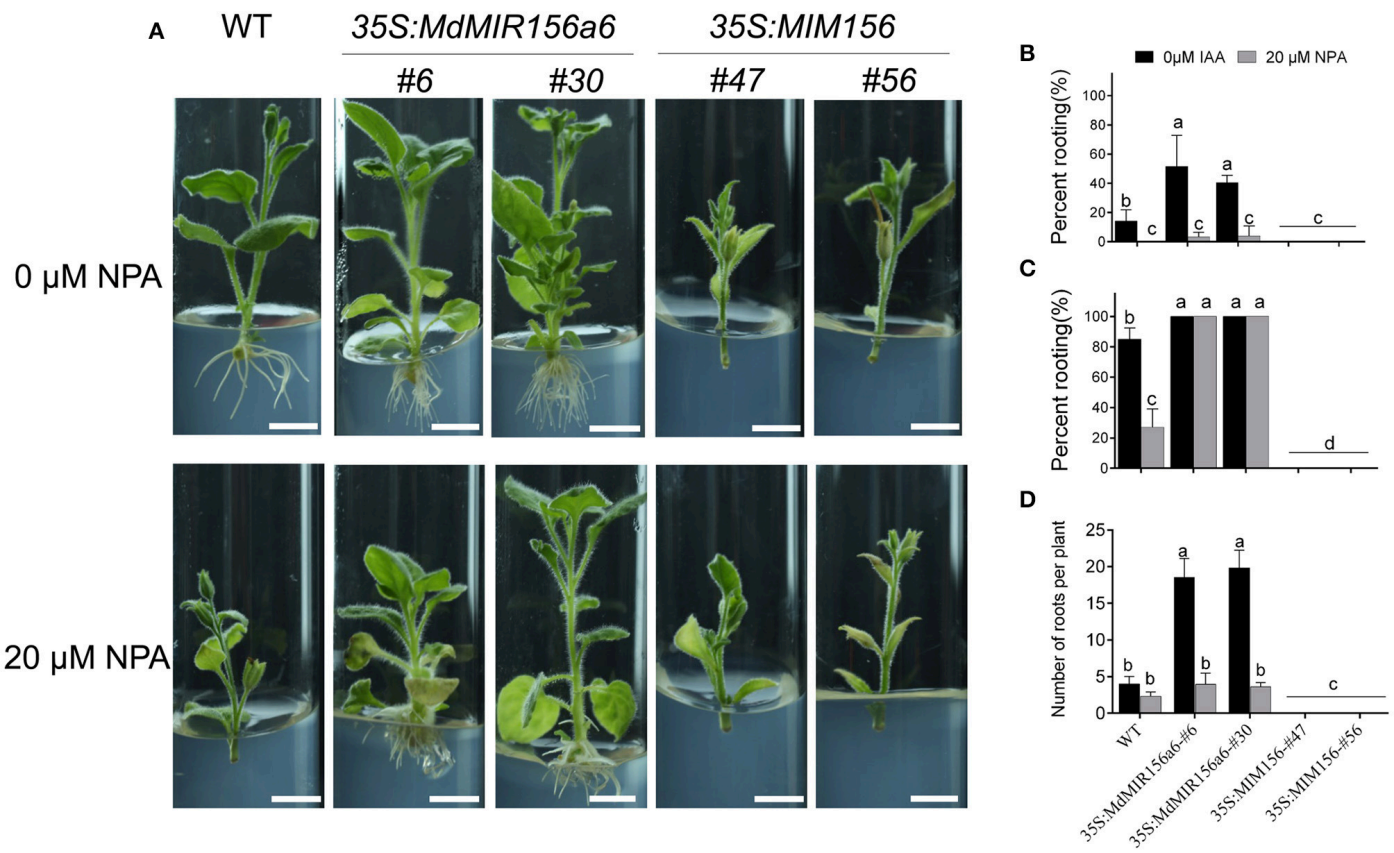
Effects of NPA on adventitious root formation in *35S:MdMIR156a6* and *35S:MIM156* tobacco plants. **(A)** Phenotypes of 13-day-old plants (WT, *35S:MdMIR156a6*, and *35S:MIM156*) grown on MS medium with 20 μM NPA. Scale bars = 15 mm. **(B,C)** Percent rooting of tobacco stem cuttings grown on MS medium with 20 μM NPA treatment for 7 and 14 d, respectively. **(D)** Adventitious root numbers per cutting. Bars show *SD* with three biological replicates; *n* = 10 cuttings in each replicate. The statistical analysis was performed by Duncan's multiple range test at level *p* ≤ 0.05. The different lowercase letter indicate significant differences.

Treatments with 20 μM NPA significantly inhibited the adventitious root formation in wild-type (Supplementary Figure 3); adventitious rooting was obviously delayed and adventitious root number was reduced in both wild-type and *35S:MdMIR156a6* plants treated with 20 μM NPA (Figure 6A). Indeed, rooting percent and root number in *35S:MdMIR156a6* plants were still consistently higher than that in wild-type (Figures 6B,C).

### *PIN* genes are not altered by miR156 expression

**Figure 7:**
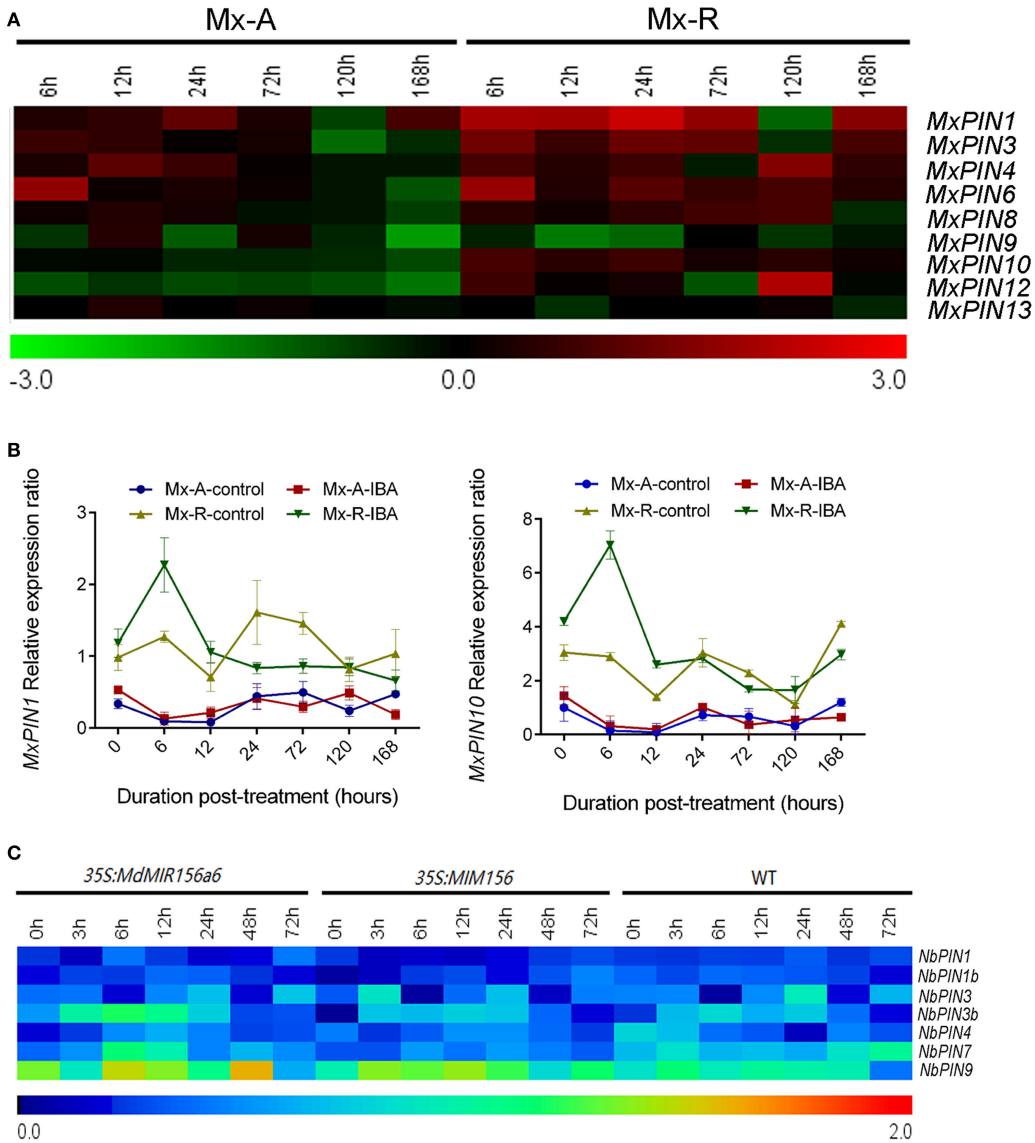
Expression profiles of *PINs* during adventitious rooting in *Malus xiaojinensis* and transgenic *Nicotiana benthamiana*. All the results of semi-quantitative RT-PCR were quantified using the ImageJ software. Log-transformed values of the relative expression levels of *MxPIN* family genes under IBA treatment compared to controls were used for hierarchical cluster analysis with MeV 4.8.1. The color scale represents relative expression levels with red denoting up-regulation and green denoting down-regulation. The relative expression levels of *NbPIN* genes compared to *NbEF1*α were used for hierarchical cluster analysis with MeV 4.8.1. Sampling times are indicated at top of the figure. **(A)** Expression profiles of *MxPIN* genes in stem bark of Mx-A and Mx-R during adventitious root formation process after IBA treatment (original results show in Supplementary Figure 4). **(B)** Quantitative RT-PCR analysis of the expression dynamics of *MxPIN1* and *MxPIN10* in stem bark from Mx-A and Mx-R during adventitious root formation after IBA treatment. Bars show *SD* from three biological replicates. **(C)** *NbPIN* genes expression patterns in WT, *35S:MdMIR156a6*, and *35S:MIM156* transgenic tobacco stems during the adventitious rooting process (original results show in Supplementary Figure 5).

The expression levels of *MxPIN3, MxPIN4, MxPIN6, MxPIN8, MxPIN9, MxPIN12*, and *MxPIN13* did not show obvious differences between Mx-A and Mx-R leafy cuttings during the adventitious root-induction process (Figure 7A and Supplementary Figure 4). The expression of *MxPIN2, MxPIN5, MxPIN7*, and *MxPIN11* was not detected in any of the RNA samples tested. As shown in Supplementary Figure 4, the expression of *MxPIN1* and *MxPIN10* was higher in Mx-R than in Mx-A with or without IBA treatment. Both *MxPIN1* and *MxPIN10* expression was induced more than 2-fold after 6 h in the presence of IBA treatment in Mx-R, but not in Mx-A (Figure 7B). The relatively high expression level of *MxPIN1* and *MxPIN10* may contribute to adventitious rooting competitive in Mx-R cuttings. However, the relationship between miR156 and *PIN* gene expression was not robust in transgenic tobacco; no distinct changes were detected in the expression of any *NbPIN* members between wild-type, *35S:MdMIR156a6*, and *35S:MIM156* tobacco plants (Figure 7C).

### *ARF* genes expression did not change with miR156 levels

**Figure 8:**
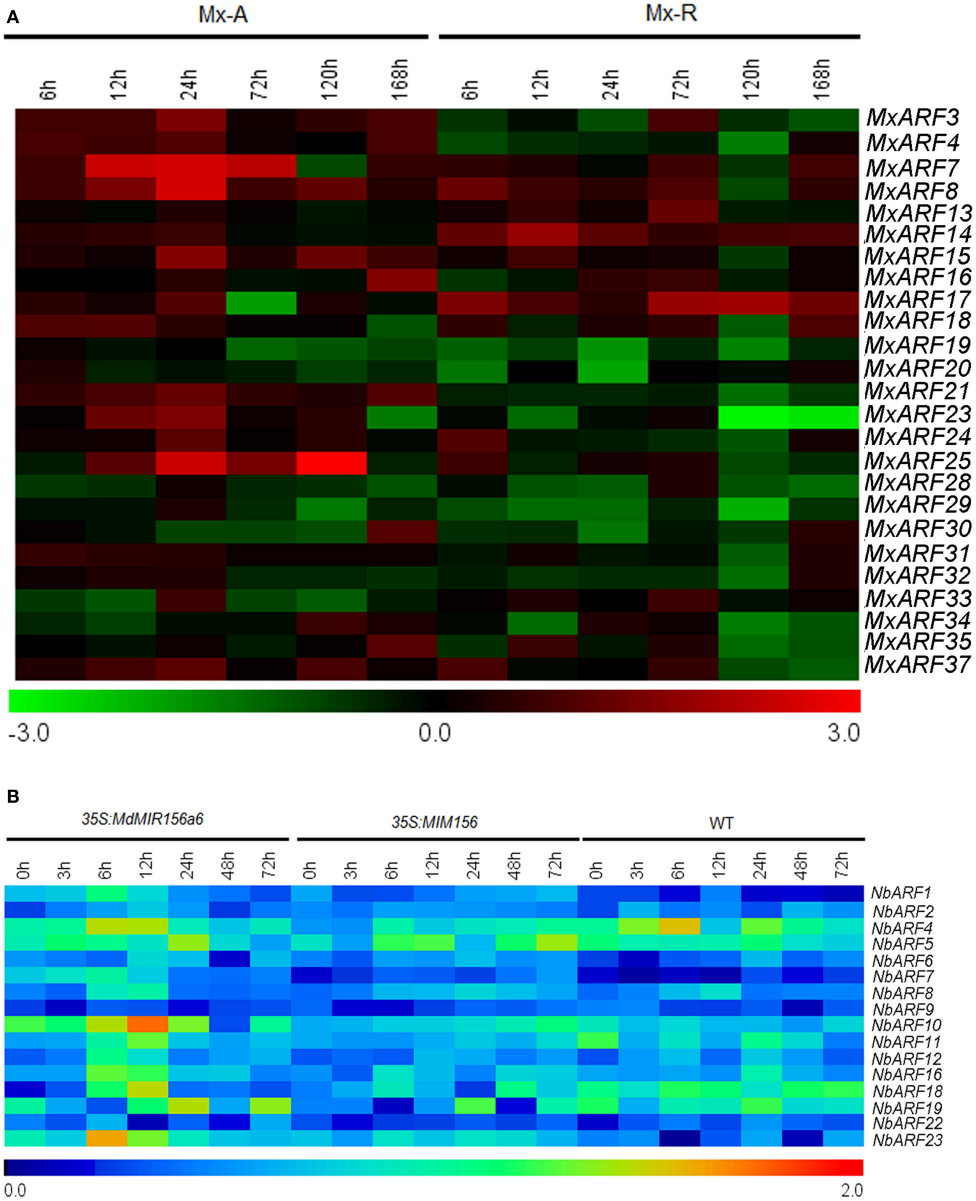
Expression profiles of *ARFs* during adventitious rooting in *Malus xiaojinensis* and transgenic *Nicotiana benthamiana*. All the results of semi-quantitative RT-PCR were quantified using the ImageJ software. Log-transformed values of the relative expression levels of *MxARF* family genes under IBA treatment compared to controls were used for hierarchical cluster analysis with MeV 4.8.1. The color scale represents relative expression levels with red denoting up-regulation and green denoting down-regulation. The relative expression levels of *NbARFs* genes compared to *NbEF1*α were used for hierarchical cluster analysis with MeV 4.8.1. Sampling times are indicated at top of the figure. **(A)** Expression profiles of *MxARF* genes in stem bark of Mx-A and Mx-R during adventitious root formation process after IBA treatment (original results show in Supplementary Figure 6). **(B)** *NbARF* genes expression patterns in WT, *35S:MdMIR156a6*, and *35S:MIM156* transgenic tobacco stems during the adventitious rooting process (original results show in Supplementary Figure 7).

The *MxARF4, MxARF7, MxARF14, MxARF15, MxARF16, MxARF17, MxARF19, MxARF20*, and *MxARF28* genes showed higher expression in Mx-A than in Mx-R cuttings with or without IBA treatment during the adventitious root formation process (Supplementary Figure 6). In contrast, expression of *MxARF3* and *MxARF8* was higher in Mx-R than in Mx-A cuttings (Supplementary Figure 6). *MxARF21, MxARF23* and *MxARF25* were down regulated in plants treated with IBA in Mx-R but not Mx-A cuttings (Figure 8A). However, no distinct changes were detected in 16 putative *NbARF* gene family members between wild-type, *35S:MdMIR156a6*, and *35S:MIM156* tobacco plants during the adventitious rooting process (Figure 8B). This indicates that miR156 may not be associated with the expression of *ARF* transcription factors during the regulation of adventitious root formation.

### *RTCS*-like gene expression varied with miR156 levels

**Figure 9:**
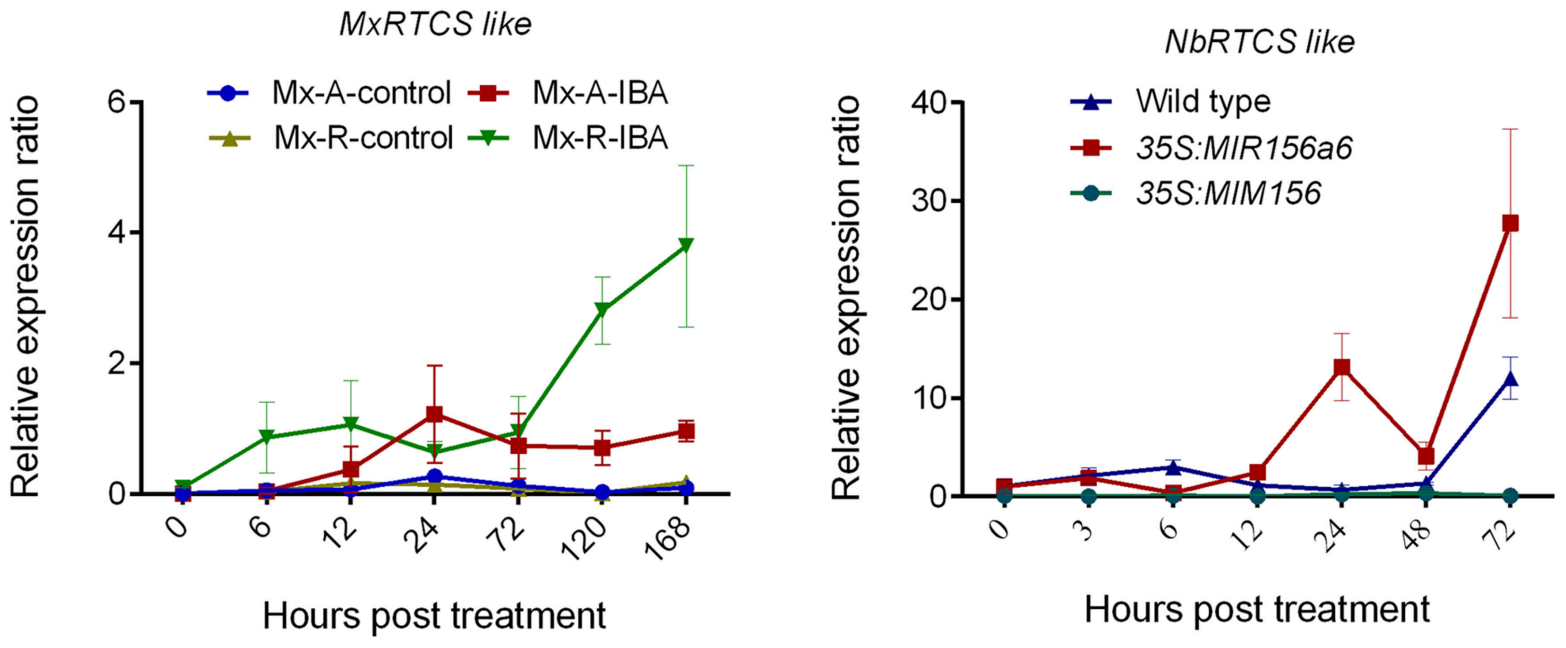
Quantitative RT-PCR analysis the expression profiles of *MxRTCS-*like and *NbRTCS-*like genes during adventitious root formation in *Malus xiaojinensis* and transgenic *Nicotiana benthamiana*. Bars show *SD* from three biological replicates.

In *M. xiaojinensis*, the *MxRTCS-*like gene was up-regulated 6–24 h after IBA treatment in both Mx-A and Mx-R cuttings, but the maximum induction of *MxRTCS* was observed in Mx-R cuttings at 120–168 h, which was 4-fold higher than that measured in Mx-A cuttings (Figure 9). Similarly, the expression of *NbRTCS* was also significantly induced 24–72 h after subculture in hormone-free medium in *35S:MdMIR156a6* plants, but was delayed 72 h subculture in hormone-free medium in wild-type. However, the expression of *NbRTCS* did not change throughout the experiment in *35S:MIM156* plants (Figure 9).

### Response in *MxSPL* gene expression to miR156 levels

**Figure 10:**
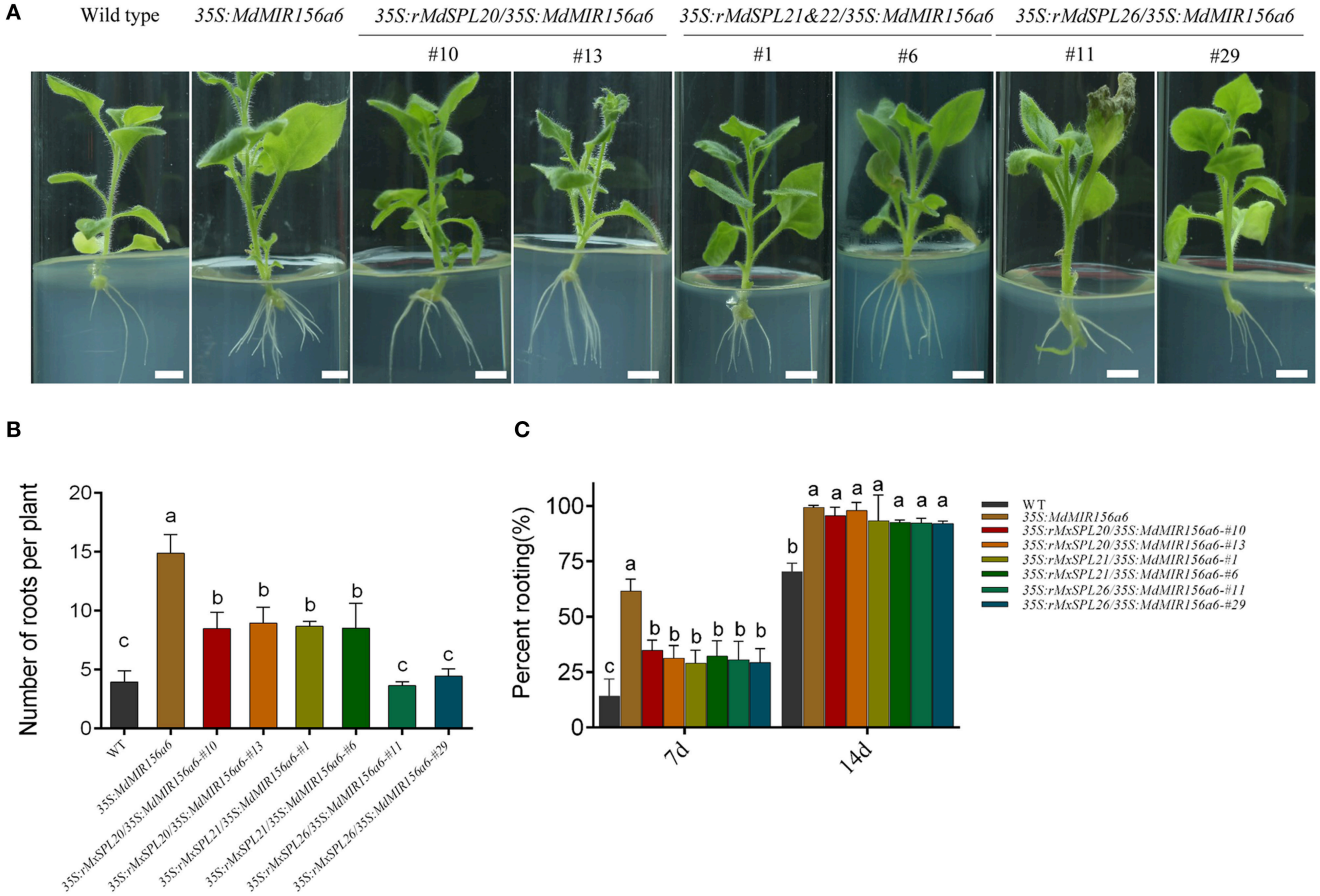
Role of *MxSPL20, 21*&*22*, and *26* genes during adventitious root formation in transgenic *Nicotiana benthamiana*. **(A)** Adventitious root formation of *35S:rMxSPL*/*35S:MdMIR156a6* plants. Scale bars = 1 cm. **(B,C)** Quantitative analysis of rooting ability of tobacco stem cuttings. **(B)** Adventitious root numbers per cutting were counted after 14 d on MS medium. **(C)** Percent rooting was investigated after 7 and 14 d of growth on MS medium. Bars show *SD* with three biological replicates; *n* = 5 for each replicate. The statistical analysis was performed by Duncan's multiple range test at level *p* ≤ 0.05. The same lowercase letter indicate no significant differences.

Of the 13 putative miR156-regulated *SPL* gene family members in apple genome, nine were actively expressed in both Mx-J and Mx-A plant cuttings, but did not include *MxSPL3, MxSPL10, MxSPL11*, and *MxSPL12*. To investigate which *M. xiaojinensis SPL* gene family member was involved in adventitious root formation, mutants of *SPL* genes, indicated here as resistant *SPLs* (*rSPLs*), were designed to no longer be targeted by miR156 (Supplementary Figure 8; Schwab et al., 2005). *MxrSPL4a*&*4b, MxrSPL18, MxrSPL19, MxrSPL20, MxrSPL21*&*22, MxrSPL24*, and *MxrSPL26*, driven by CaMV 35S promoter, were transformed into *35S:MdMIR156a6* transgenic tobacco plants (Supplementary Figure 9). In independent bivalent transgenic lines, *35S:rSPL4a*&*4b*/*35S:MdMIR156a6, 35S:rSPL18*/*35S:MdMIR156a6, 35S:rSPL19*/*35S:MdMIR156a6*, and *35S:rSPL24*/*35S:MdMIR156a6* the adventitious rooting rate and adventitious root number did not significantly differ from that of plants expressing *35S:MdMIR156a6* (Supplementary Figure 10), indicating that *MxSPL4a*&*4b, MxSPL18, MxSPL19*, and *MxSPL24* are not involved in adventitious rooting. In contrast, the bivalent transformants *35S:rSPL20*/*35S:MdMIR156a6, 35S:rSPL21*&*22*/*35S:MdMIR156a6*, and *35S:rSPL26*/*35S:MdMIR156a6* exhibited reduced adventitious rooting ability (Figure 10A). *35S:rSPL20*/*35S:MdMIR156a6* and *35S:rSPL21*&*22*/*35S:MdMIR156a6* produced significantly fewer adventitious roots than *35S:MdMIR156a6* plants, but still more than wild-type plants (Figure 10B). The adventitious root number in *35S:rSPL26*/*35S:MdMIR156a6* plants was significantly fewer than that in *35S:MdMIR156a6* plants (Figure 10B). In addition, the adventitious root development rate in *35S:rSPL20*/*35S:MdMIR156a6, 35S:rSPL21*&*22*/*35S:MdMIR156a6*, and *35S:rSPL26*/*35S:MdMIR156a6* plants were similar as that measured in wild-type, but slower than that observed in *35S:MdMIR156a6* plants 7 d after culture on MS medium (Figure 10C). These results indicate that *MxSPL20, MxSPL21*&*22*, and *MxSPL26* not only affected adventitious root number but also delayed rooting rate.

**Figure 11:**
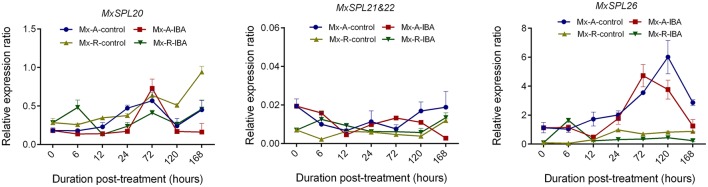
Expression profile of *MxSPL20, 21*&*22*, and *26* genes during adventitious root formation in *Malus xiaojinensis* leafy cuttings. RNA was extracted from Mx-A and Mx-R stem bark after IBA treatment at serial time-points. Relative expression was measured using quantitative real time PCR normalized to *MxEF1*α. Bars show *SD* from three biological replicates.

The *MxSPL20, MxSPL21*&*22*, and *MxSPL26* expression profile was detected at 0, 6, 12, 24, 72, 120, and 168 h after IBA treatment (Figure 11). There were no obviously differences in the expression of *MxSPL20* and *MxSPL21*&*22* between Mx-A and Mx-R cuttings; however, the expression of *MxSPL26* was significantly higher in Mx-A than in Mx-R cuttings, indicating *MxSPL26* could be a key *SPL* family member involved in adventitious rooting of leafy cuttings.

## Discussion

Although, it appears that juvenile phase softwood cuttings are much easier to root than the adult ones, there is little data detailing the mechanism of these differences. The expression level of miR156 was decreased during juvenile to adult phase change (Du et al., 2015; Ji et al., 2016). However, the effect of miR156 on adventitious root formation barely rated a mention in previous reports (Zhang et al., 2011; Feng et al., 2016; Massoumi et al., 2017). Although, miR156-targeted *SPLs* are known to control varied physiological and developmental processes (Wang et al., 2008; Shikata et al., 2009; Yu et al., 2010, 2015; Gou et al., 2011), with which the miR156-targeted SPL gene member is associated with adventitious rooting and how miR156 interacts with other rooting regulatory factors such as IAA are so far not fully understood to date. In the present study, we found that the high miR156 expression was required for adventitious roots formation in an apple rootstock, *M. xiaojinensis*. We also confirmed the involvement of *MxSPL26* in inhibiting adventitious root formation.

### Auxin and high miR156 expression level are both necessary for adventitious rooting

For some rooting recalcitrant woody plants, juvenility is necessary for efficient adventitious rooting. In general, cuttings from juvenile nodes root more readily than cuttings from adult nodes in woody plants (Greenwood, 1995). Indeed, miR156 expression levels were significantly higher in Mx-J and Mx-R cuttings than Mx-A cuttings (Figure 2), and the rooting ability of Mx-R and Mx-J cuttings were consistently higher than that of Mx-A cuttings (Figure 1). A significant decrease in miR156 expression was observed in shoots taken from 1.4 m trunk above ground compared to shoots taken from below 1.4 m in *M. xiaojinesis* seedlings (Ji et al., 2016). In our previous data, the micro-shoots from adult phase *M. xiaojinnesis* explants were rejuvenated successfully after 15 passages of *in vitro* subculture, marked by the elevated expression of miR156 expression in leaves of the micro-shoots, and coupled with recovered adventitious rooting ability and leaf lobes (Xiao et al., 2014).

When miR156 level was manipulated via transformation with a *35S:MIR156* construct in tomato, tobacco, or *Arabidopsis*, the adventitious rooting increased (Zhang et al., 2011; Feng et al., 2016; Massoumi et al., 2017). In the present study, the miR156 expression level was manipulated in transgenic tobacco to analyze how miR156/SPL modules are involved in adventitious rooting. Consistent with the previous reports, the adventitious rooting capacity also varied with *35S:MIM156* and *35S:MdMIR156a6* overexpressing transgenic tobacco plants (Figure 3). However, the uncoupling of miR156 expression and adventitious root formation was reported in *E. grandis* (Levy et al., 2014); therefore, miR156 may be necessary but not sufficient for adventitious rooting in woody plants. Except for high expression of miR156, auxin was also integrant for adventitious root formation. In absence of IBA treatment, even Mx-J and Mx-R leafy cuttings exhibit adventitious root defects (Figure 1). In agreement to this result, the adventitious rooting ability was reduced in all tobacco lines upon treatment with the polar auxin transport inhibitor NPA (Figure 6).

### miR156 affects adventitious root formation independently from *PIN* and *ARF* genes expression

Auxin and miR156 are both involved in adventitious root development (Zhang et al., 2011; Feng et al., 2016; Steffens and Rasmussen, 2016; Massoumi et al., 2017). However, the interaction of miR156 and auxin signaling pathway is unexplored during adventitious root development. In comparison to the wild-type samples, *NbPINs* and *NbARFs* expressions was not substantially modulated in *35S:MdMIR156a6* and *35S:MIM156* plants (Figures 7, 8), indicating that miR156 promoted adventitious root formation independently from changing *PIN* and *ARF* genes expression. These results are supported by the transcriptome data, which show that *PIN* and *ARF* genes in *Medicago sativa* are not significantly differentially expressed between miR156 overexpression and wild-type plants (Gao et al., 2016). Similarly, the auxin response was not changed in either *Pro35S:MIR156* or *Pro35S:MIM156 Arabidopsis*, indicating that miR156 does not modulate the auxin response during regulating shoot regeneration (Zhang T. Q. et al., 2015). Collectively, these findings suggest that miR156 does not modulate the early auxin response.

Conversely, auxin can induce the expressions of two *MIR156* genes and two *SPL* genes during lateral root development in transgenic *Arabidopsis* (Yu et al., 2015). GUS staining revealed that *MIR156B* was specifically expressed in primary and lateral root primordia, *MIR156D* was especially active in primary and lateral root tip, and two *SPL* genes were also highly or specifically expressed in root (Yu et al., 2015). Hence, auxin inducing *MIR156* and *SPL* expression may be tissue/organ specific during lateral root development. Our results revealed that the expression level of *MxSPL26* or *MxSPL20* or *MxSPL21*&*22* was not obviously affected under IBA treatment (Figure 11). Similarly, miR156 expression was not affected by IBA application during the induction of adventitious root development in both juvenile and adult *E. grandis* stem cuttings (Levy et al., 2014). Thus, miR156/SPL modules were not downstream targets of auxin during adventitious root formation.

**Figure 12:**
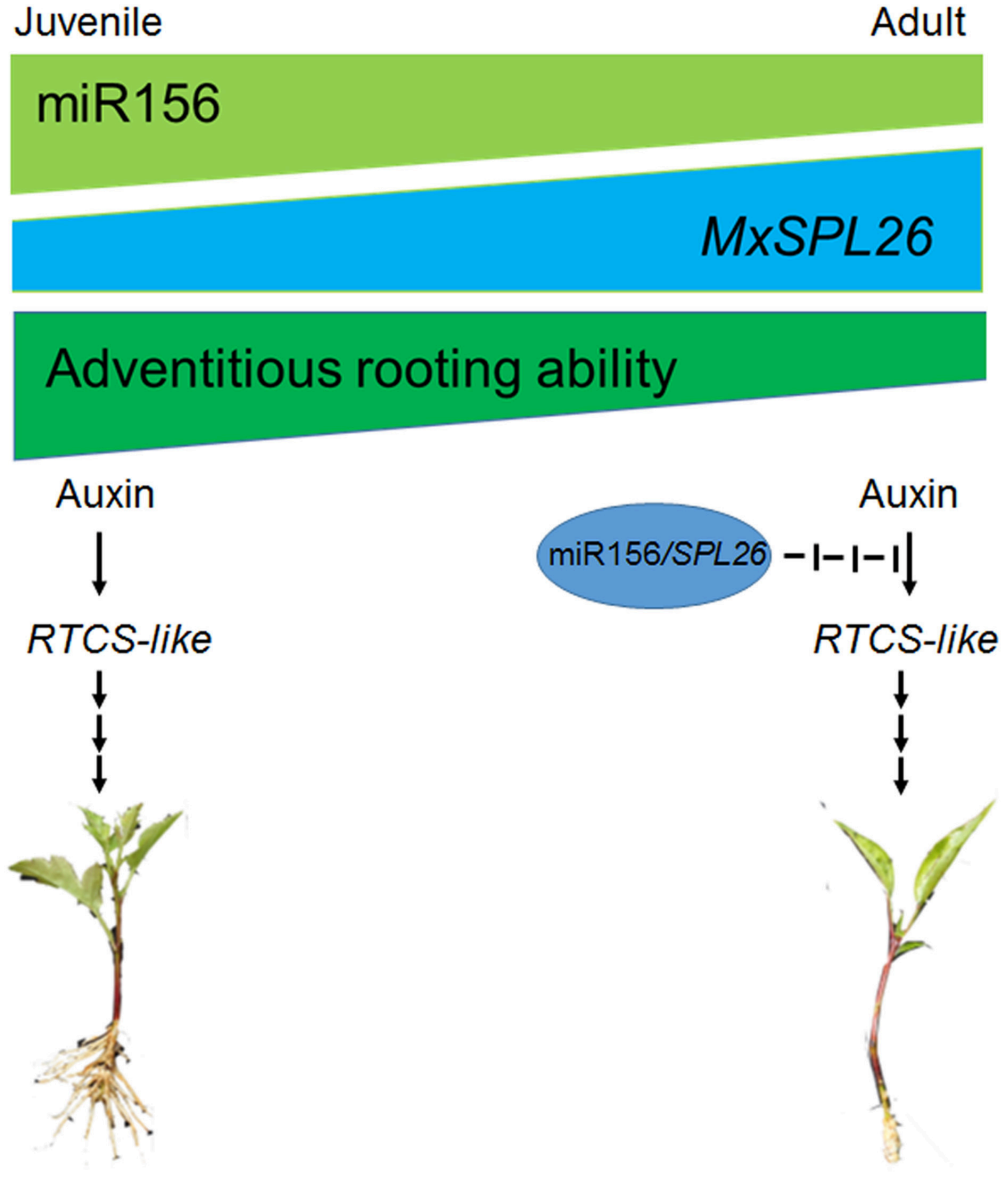
Illustration of role of juvenility and auxin in triggering adventitious rooting. In adult leafy cuttings, decline of miR156 caused high expression level of SPL26. This modules may inhibit transcript abundances of *RTCS*-like gene induced by auxin, thereby reducing the adventitious rooting capacity in adult plants.

*Rtcs* and *Rtcl* in maize are orthologs of *CRL1* in rice (*O. sativa*) and of *AtLBD29* in *A. thaliana*, and all of these genes are involved in the initiation of adventitious root formation and are targets of the ARF gene family (Inukai et al., 2005; Taramino et al., 2007; Liu J. C. et al., 2014). Although *MxRTCS-*like gene expression was induced in both Mx-A and Mx-R cuttings after IBA treatment, the distinct fold-change in expression occurred only in Mx-R cuttings (Figure 9). In agreement with these finding, the *NbRTCS* expression was significantly induced in *35S:MdMIR156a6* transgenic tobacco plants, but no substantial changes were detected in *35S:MIM156* transformants (Figure 9). These data suggest that high expression level of miR156 is required for auxin inducing expression of *RTCS*-like during adventitious root formation in *M. xiaojinesis* and transgenic tobacco. Therefore, we speculate that decline of miR156 expression in adult phase leafy cuttings may inhibit transcript abundance of *RTCS*-like gene induced by auxin, thereby reducing the adventitious rooting capacity (Figure 12). The histological features showed that Mx-A leafy cuttings and *35S:MIM156* transgenic tobacco plants exhibited defect in adventitious root primordia initiation (Figures 1E,4), which provided an evidence for above mentioned speculation. In agreement with our observations, maize mutant *rtcs* and rice mutant *rtcl* both failed in adventitious root primordia initiation (Inukai et al., 2005; Muthreich et al., 2013). However, the molecular mechanism underlying miR156 modulate auxin induced *RTCS*-like gene expression remains unknown.

Although, the expressions of *PIN* and *ARF* genes were independently from the miR156 expression level, the response of *MxPIN1* and *MxPIN10* gene expression to auxin treatment differed in Mx-R and Mx-A samples. The different responses of *PIN* family members to auxin treatment between Mx-R and Mx-A cuttings may be caused by other developmental signals, such as glutathione content. In apple seedlings, the glutathione content and glutathione/glutathione disulfide ratio were much higher in the juvenile phase than in the adult phase, and modulating glutathione content caused concomitant changes in miR156 expression levels (Du et al., 2015). It has been proved that the reduction of glutathione availability by L-buthionine-(*S,R*)-sulfoximine (BSO) treatment reduced the expression of *PIN1, PIN2*, and *PIN3* in *Arabidopsis* (Bashandy et al., 2010).

In addition, *MxARF4, MxARF7, MxARF14, MxARF15, MxARF16, MxARF17, MxARF19, MxARF20*, and *MxARF28* showed relative higher expression levels in Mx-A than in Mx-R cuttings during adventitious root formation (Supplementary Figure 6). Interestingly, the peptide sequences of these ARFs, except for ARF20, are enriched in serine, proline, and leucine in their middle regions (Supplementary Figure 11). *Arabidopsis* ARFs with this middle region are usually transcriptional repressors (Guilfoyle and Hagen, 2007). The differential expression of *MxPIN* and *MxARF* might partly cause Mx-A recalcitrant to root formation.

### *MxSPL26* is the key target gene in miR156 regulation of adventitious rooting in *M. xiaojinensis*

MiR156 and its targeted *SPL* genes were demonstrated to control plant phase transition and related traits such as cell size and number, trichome development, anthocyanin synthesis, leafy morphology, flowering time, and lateral root development (Wang et al., 2008; Shikata et al., 2009; Yu et al., 2010, 2015; Gou et al., 2011). Yet, the function of *SPL* genes involving in adventitious rooting are largely unexplored. It was reported that hypocotyls of *spl2/9/11/13/15* mutants, as well as plants transformed with *35S:MIR156A*, produced the same number of adventitious roots as wild-type plants (Xu et al., 2016). However, it is not clear which *SPL* gene involved in regulating adventitious rooting in *Arabidopsis*, because the expression level of miR156 targeted *SPLs* had no significantly difference among s*pl2/9/11/13/15* mutants, *35S:MIR156A* plants and wild-type young seedlings (Xu et al., 2016). Here, *MxSPL26* was experimentally confirmed to be involved in inhibiting adventitious root formation most possibly in function redundancy with *SPL20* and *SPL21*&*22*, but *SPL26* seemed to play a major role. The expression of *MxSPL26* was constantly higher than that of *MxSPL20* and *MxSPL21*&*22* in *M. xiaojinensis* cuttings. *MxSPL26* relative expression exhibited a 5- to 10-fold increase in stem bark from Mx-A cuttings than in that from Mx-R after 72~120 h from plugging into the media, but no obvious differences in the expression of *MxSPL20* and *MxSPL21*&*22* genes were found between Mx-A and Mx-R (Figure 11). The number of roots per plant was significantly reduced in all the *35S:rMxSPLs/35S:MdMIR156a6* lines but *MxSPL26* resistant form transgenic tobacco lines produced the least number of adventitious roots compared with *rMxSPL20, rMxSPL21*&*22* and *35S:MdMIR156a6* transgenic lines (Figure 10 and Supplementary Figure 10). According to the phylogenetic tree, *MxSPL20* and *MxSPL21*&*22* exhibited close evolutionary, but quite far phylogenetic relationship from *MxSPL26* (Supplementary Figure 12). Notably, although SPL3, SPL9, and SPL10 were all involved in repressing *Arabidopsis* lateral root development, SPL10 seemed to play a major role (Yu et al., 2015). Similarly, *AtSPL3* and *AtSPL9* exhibited close evolutionary, but quite far phylogenetic relationship from *AtSPL10* (Supplementary Figure 12).

In conclusion, in semi-lignified leafy cuttings of *M. xiaojinensis*, a relatively higher expression of the miR156 is necessary for adventitious root formation. MiR156 functions via its target gene *MxSPL26* and rooting-related genes such as *MxRTCS*-like; but acts independently of *MxPIN* or *MxARF* family members in response to auxin. These results provided a potential strategy for the improvement of the adventitious rooting ability of perennial woody plants via manipulating miR156 and *SPL* gene expression.

## Author contributions

XZ, YW, TW, XzX, and ZH prepared the plant materials and designed the experiments. XzX, XL, and XH conducted the experiments. XzX took the photographs. XzX and XZ analyzed the data and XzX wrote the manuscript. All authors read and approved the manuscript.

### Conflict of interest statement

The authors declare that the research was conducted in the absence of any commercial or financial relationships that could be construed as a potential conflict of interest.

## Supplementary material

Supplementary Figure 1 Expression profiles of nine miR156 precursors were analyzed using semi-quantitative RT-PCR in *Malus xiaojinensis* stem bark.

Supplementary Figure 2 Histological features of Mx-A, Mx-J, and Mx-R leafy cuttings of *Malus xiaojinensis* before IBA treatment. Cross sections of the stems were stained with toluidine blue. Scale bars = 200 μm.

Supplementary Figure 3 NPA (1-N-naphthylphthalamic acid) concentration selected for wild type tobacco plant treatment. Tobacco plants were transferred to MS medium with 5, 20, or 80 μM NPA for 14 days. **(A)** The phenotype and **(B)** percent rooting were evaluated. Bars show *SD* from three biological replicates; *n* = 10 individuals in each replicate. Scale bars = 15 mm.

Supplementary Figure 4 Semi-quantitative RT-PCR analysis of the expression dynamics of *MxPINs* in stem barks from Mx-A and Mx-R during adventitious root formation in *Malus xiaojinensis* under IBA treatment. *MxEF1*α was used as an internal control. The upper and lower bands represent treatment and control, respectively.

Supplementary Figure 5 *NbPIN* genes expression pattern in tobacco stems from WT, *35S:MdMIR156a6*, and *35S:MIM156* transgenic lines growing on MS medium during the adventitious rooting process. *NbEF1*α was used as an internal control.

Supplementary Figure 6 Semi-quantitative RT-PCR analysis of the expression dynamics of *MxARFs* in stem barks from Mx-A and Mx-R during adventitious root formation in *Malus xiaojinensis* under IBA treatment. *MxEF1*α was used as an internal control. The upper and lower bands represent treatment and control, respectively.

Supplementary Figure 7 *NbARF* genes expression pattern in tobacco stems from WT, *35S:MdMIR156a6*, and *35S:MIM156* transgenic lines growing on MS medium during the adventitious rooting process. *NbEF1*α was used as an internal control.

Supplementary Figure 8 Diagram of the miR156 target sites of the WT and modified version of *MxSPLs*. Capital letters indicate the amino acid sequences in *Malus xiaojinesis*.

Supplementary Figure 9 Molecular characterization of transgenic tobacco plants. **(A)** Semi-quantitative PCR analysis of *rMxSPLs* DNA and mRNA levels and **(B)** miR156 expression levels in 10-day old seedling leaves.

Supplementary Figure 10 Role of *MxSPL4a*&*4b, 18, 19*, and *24* genes during adventitious root formation in transgenic *Nicotiana benthamiana*. **(A)** Adventitious root formation in *35S:rMxSPL*/*35S:MdMIR156a6* plants. Scale bars = 1 cm. **(B,C)** Quantitative analysis of the rooting ability in tobacco stem cuttings. **(B)** Percent rooting was investigated after 7 and 14 d on MS medium. **(C)** Adventitious root numbers per cutting were counted after 14 d on MS medium. Bars show *SD* from three biological replicates; *n* = 5 plants in each individual replicate. The statistical analysis was performed by Duncan's multiple range test at level *p* ≤ 0.05; means with different letters are significantly different from each other.

Supplementary Figure 11 The MxARF family of transcription factors in *Malus domestic*. MdARF2, 11, 12, 20, 30, 33, and 36–39 have an activation domain (AD) that is enriched in glutamine (Q), serine (S), and leucine (L). The remainder of the ARFs consist of transcriptional repressors with a repression domain (RD) that is enriched in serine (S) and in some cases proline (P). All ARFs contain a conserved DNA binding domain (DBD).

Supplementary Figure 12 Phylogenetic analysis of miR156-targeted SPL between apple and *Arabidopsis*. Phylogenetic tree was constructed with SBP domain protein sequences. Phylogenetic tree was constructed using MEGA 4.0 software with the neighbor-joining (NJ) method and the bootstrap test replicated 1,000 times.

Supplementary Table 1 Primers for constructing vector.

Supplementary Table 2 PCR primers of genes in *Malus xiaojinesis*.

Supplementary Table 3 PCR primers of genes in *Nicotiana benthamiana*.
