# Supplementary figures and images for: High miR156 Expression Is Required for Auxin-Induced Adventitious Root Formation via MxSPL26 Independent of PINs and ARFs in Malus xiaojinensis
Source: Front Plant Sci. 2017 Jun 19;8:1059. doi: 10.3389/fpls.2017.01059 (PMC5474533; doi:10.3389/fpls.2017.01059)

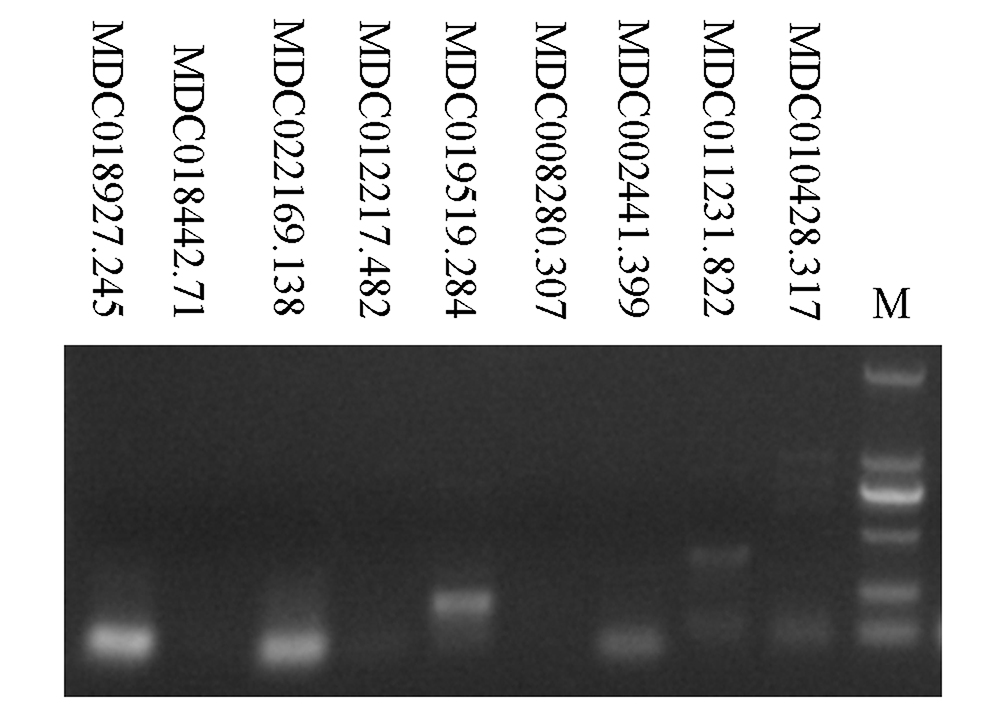

Supplement: Supplementary Figure 1 — Expression profiles of nine miR156 precursors were analyzed using semi-quantitative RT-PCR in Malus xiaojinensis stem bark. [file Image1.TIF]

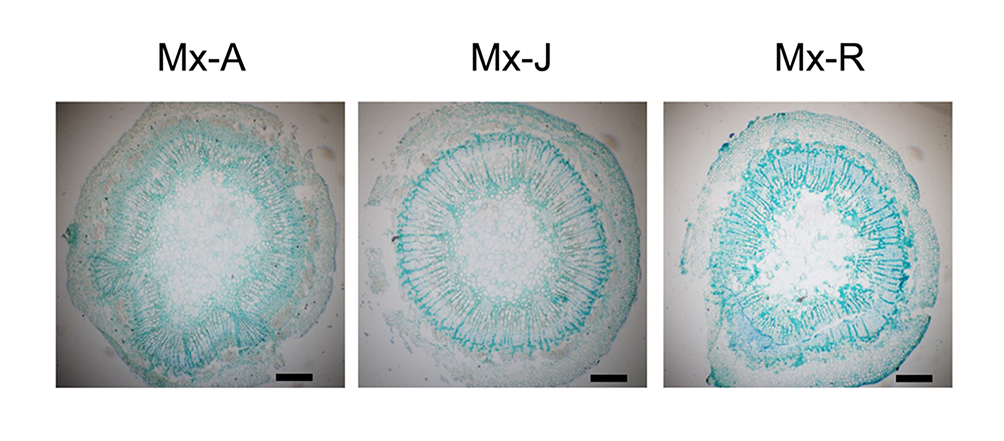

Supplement: Supplementary Figure 2 — Histological features of Mx-A, Mx-J, and Mx-R leafy cuttings of Malus xiaojinensis before IBA treatment. Cross sections of the stems were stained with toluidine blue. Scale bars = 200 μm. [file Image2.TIF]

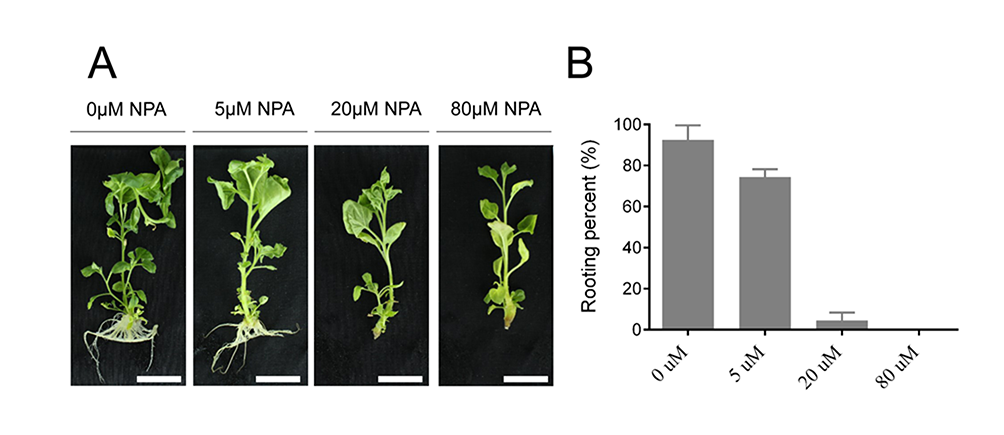

Supplement: Supplementary Figure 3 — NPA (1-N-naphthylphthalamic acid) concentration selected for wild type tobacco plant treatment. Tobacco plants were transferred to MS medium with 5, 20, or 80 μM NPA for 14 days. (A) The phenotype and (B) percent rooting were evaluated. Bars show SD from three biological replicates; n = 10 individuals in each replicate. Scale bars = 15 mm. [file Image3.TIF]

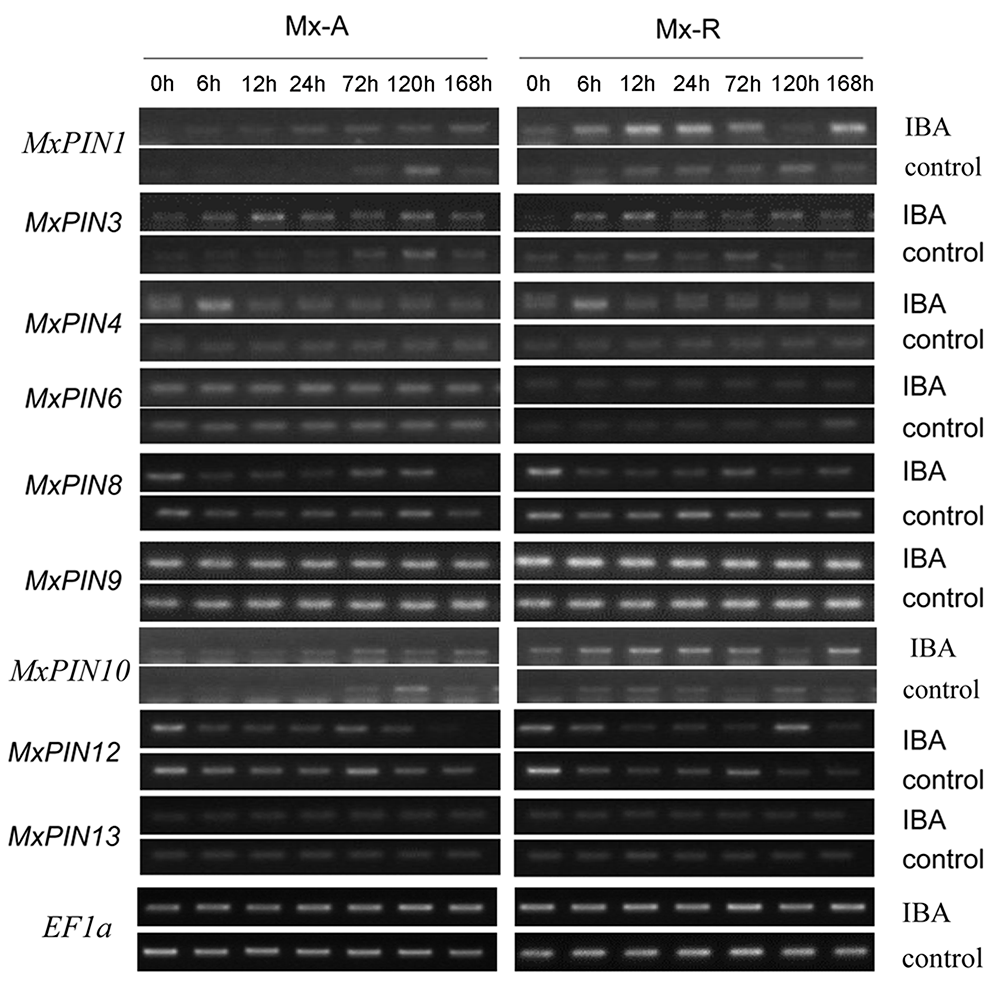

Supplement: Supplementary Figure 4 — Semi-quantitative RT-PCR analysis of the expression dynamics of MxPINs in stem barks from Mx-A and Mx-R during adventitious root formation in Malus xiaojinensis under IBA treatment. MxEF1α was used as an internal control. The upper and lower bands represent treatment and control, respectively. [file Image4.TIF]

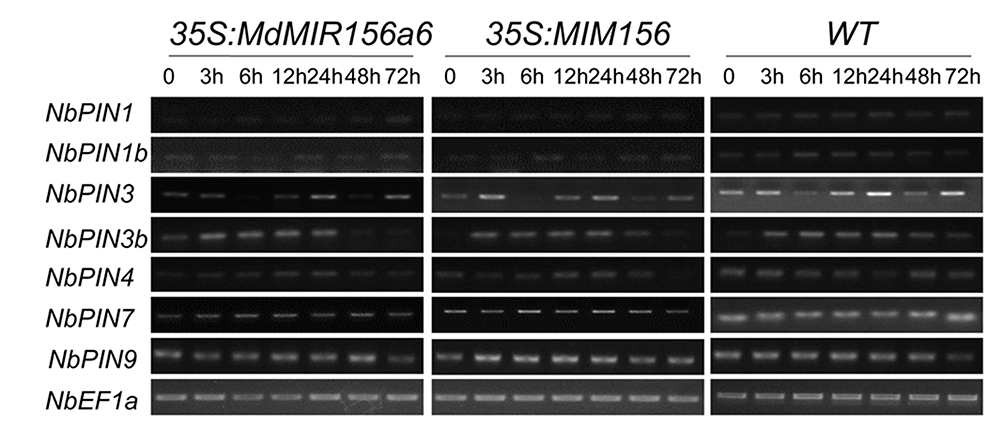

Supplement: Supplementary Figure 5 — NbPIN genes expression pattern in tobacco stems from WT, 35S:MdMIR156a6, and 35S:MIM156 transgenic lines growing on MS medium during the adventitious rooting process. NbEF1α was used as an internal control. [file Image5.TIF]

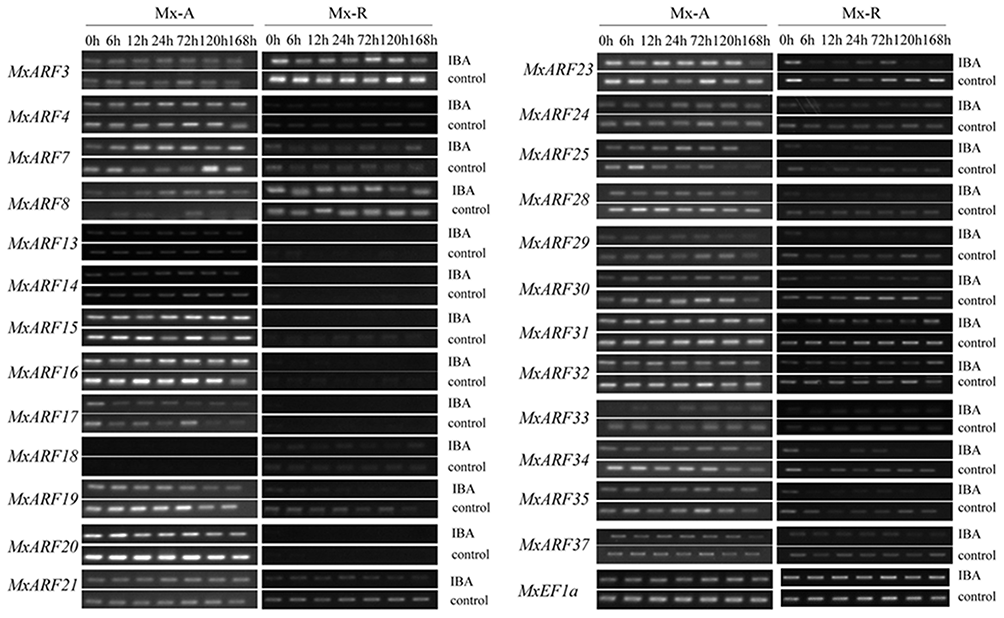

Supplement: Supplementary Figure 6 — Semi-quantitative RT-PCR analysis of the expression dynamics of MxARFs in stem barks from Mx-A and Mx-R during adventitious root formation in Malus xiaojinensis under IBA treatment. MxEF1α was used as an internal control. The upper and lower bands represent treatment and control, respectively. [file Image6.TIF]

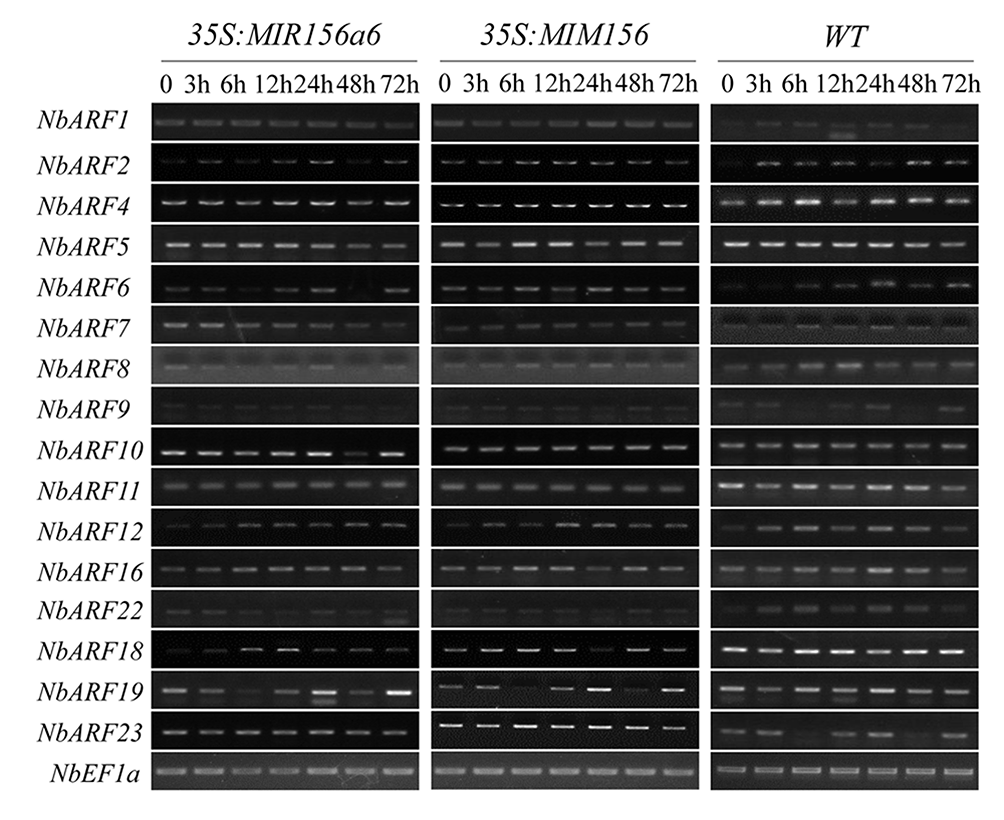

Supplement: Supplementary Figure 7 — NbARF genes expression pattern in tobacco stems from WT, 35S:MdMIR156a6, and 35S:MIM156 transgenic lines growing on MS medium during the adventitious rooting process. NbEF1α was used as an internal control. [file Image7.TIF]

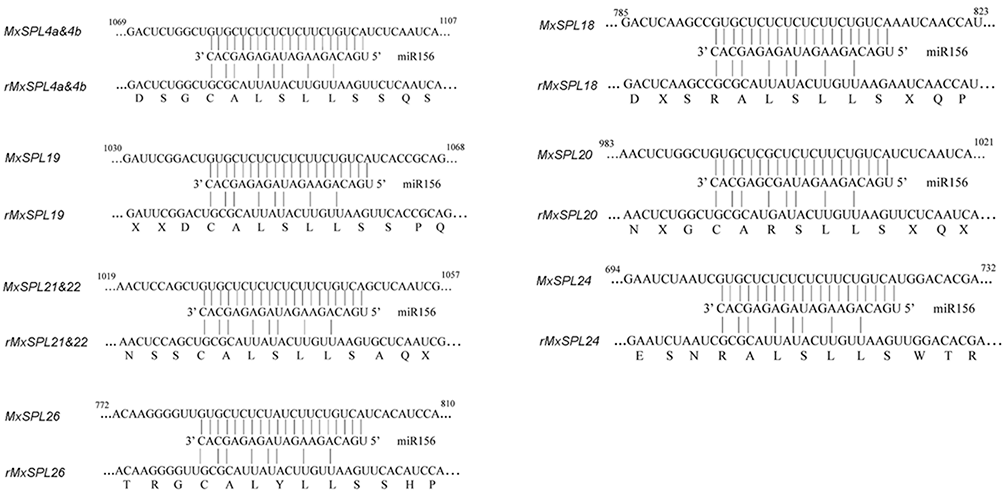

Supplement: Supplementary Figure 8 — Diagram of the miR156 target sites of the WT and modified version of MxSPLs. Capital letters indicate the amino acid sequences in Malus xiaojinesis. [file Image8.TIF]

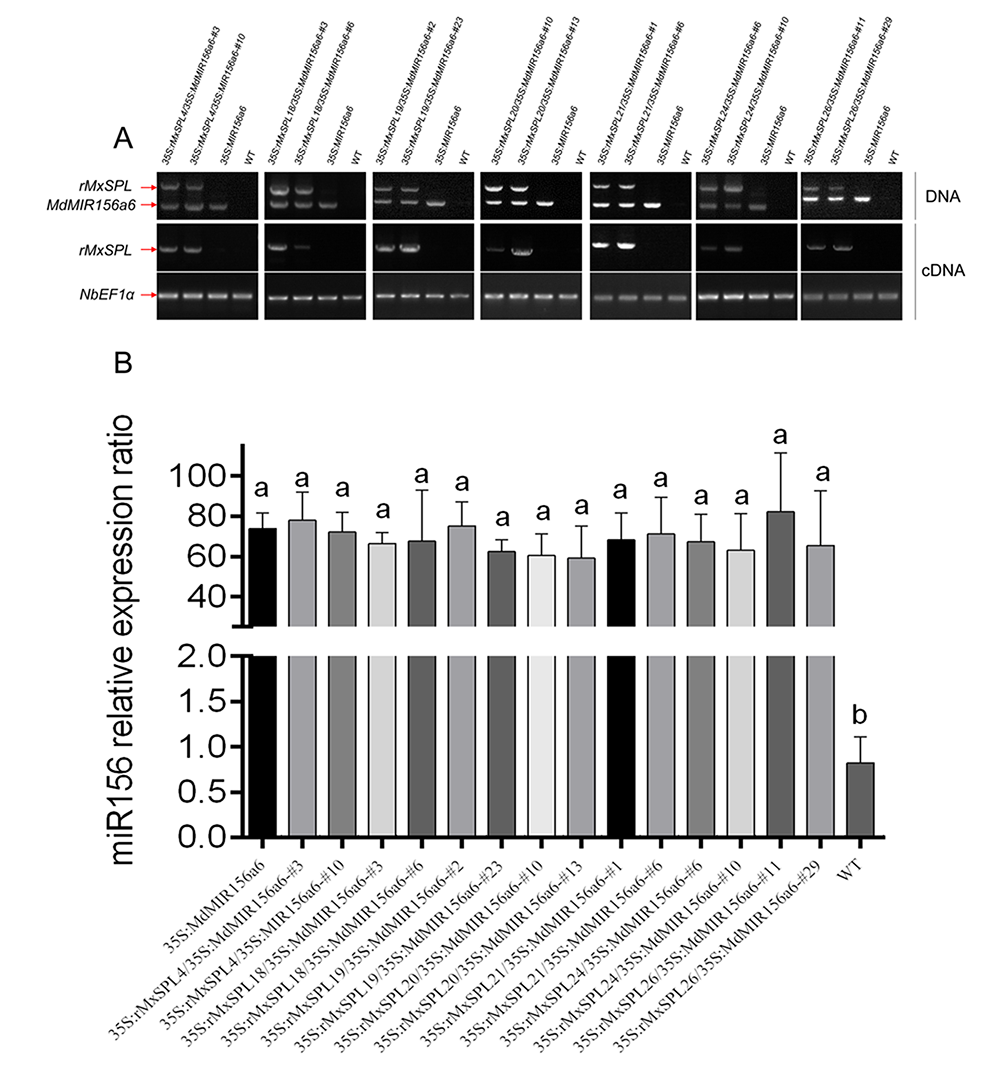

Supplement: Supplementary Figure 9 — Molecular characterization of transgenic tobacco plants. (A) Semi-quantitative PCR analysis of rMxSPLs DNA and mRNA levels and (B) miR156 expression levels in 10-day old seedling leaves. [file Image9.TIF]

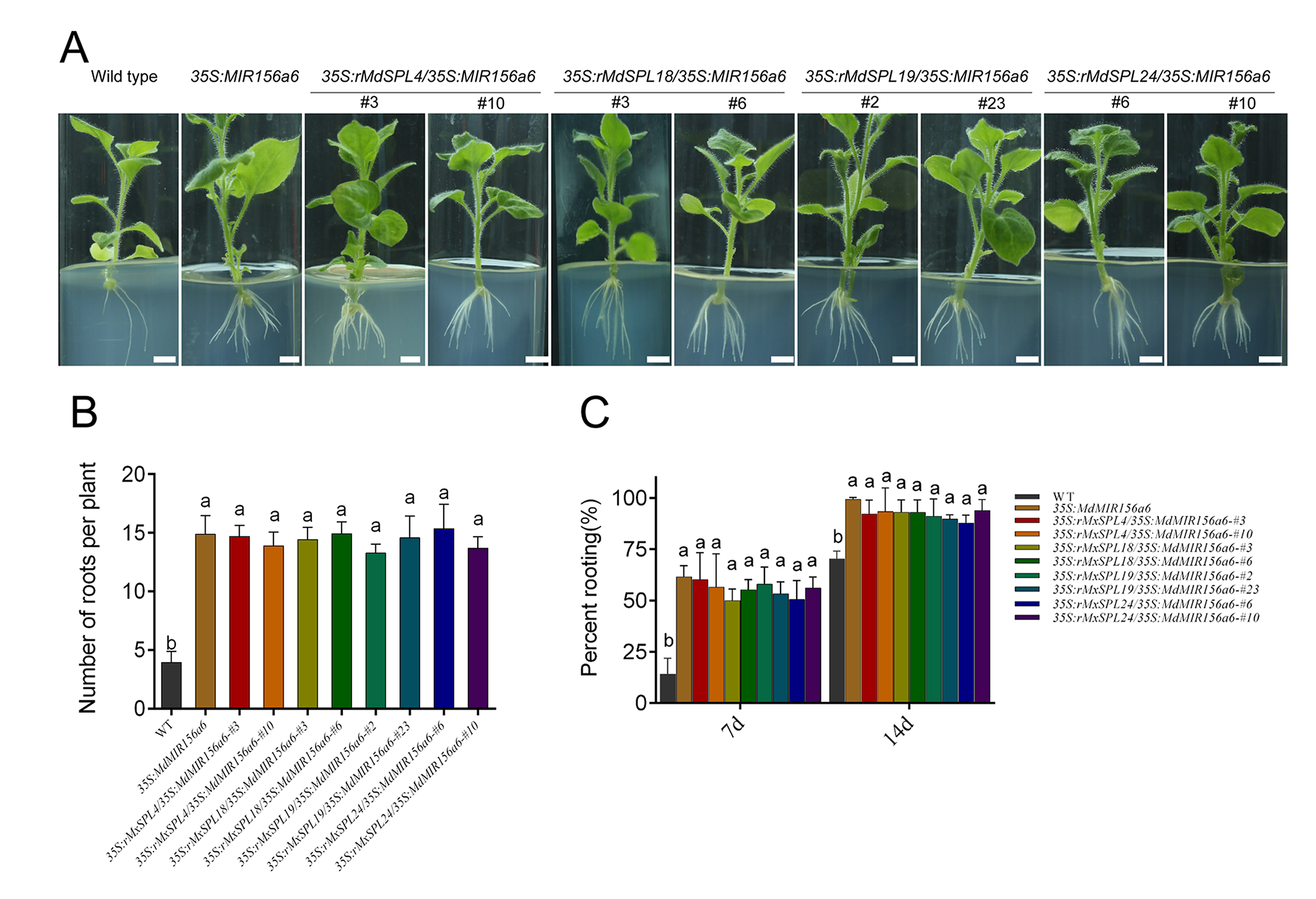

Supplement: Supplementary Figure 10 — Role of MxSPL4a&4b, 18, 19, and 24 genes during adventitious root formation in transgenic Nicotiana benthamiana. (A) Adventitious root formation in 35S:rMxSPL/35S:MdMIR156a6 plants. Scale bars = 1 cm. (B,C) Quantitative analysis of the rooting ability in tobacco stem cuttings. (B) Percent rooting was investigated after 7 and 14 d on MS medium. (C) Adventitious root numbers per cutting were counted after 14 d on MS medium. Bars show SD from three biological replicates; n = 5 plants in each individual replicate. The statistical analysis was performed by Duncan's multiple range test at level p ≤ 0.05; means with different letters are significantly different from each other. [file Image10.TIF]

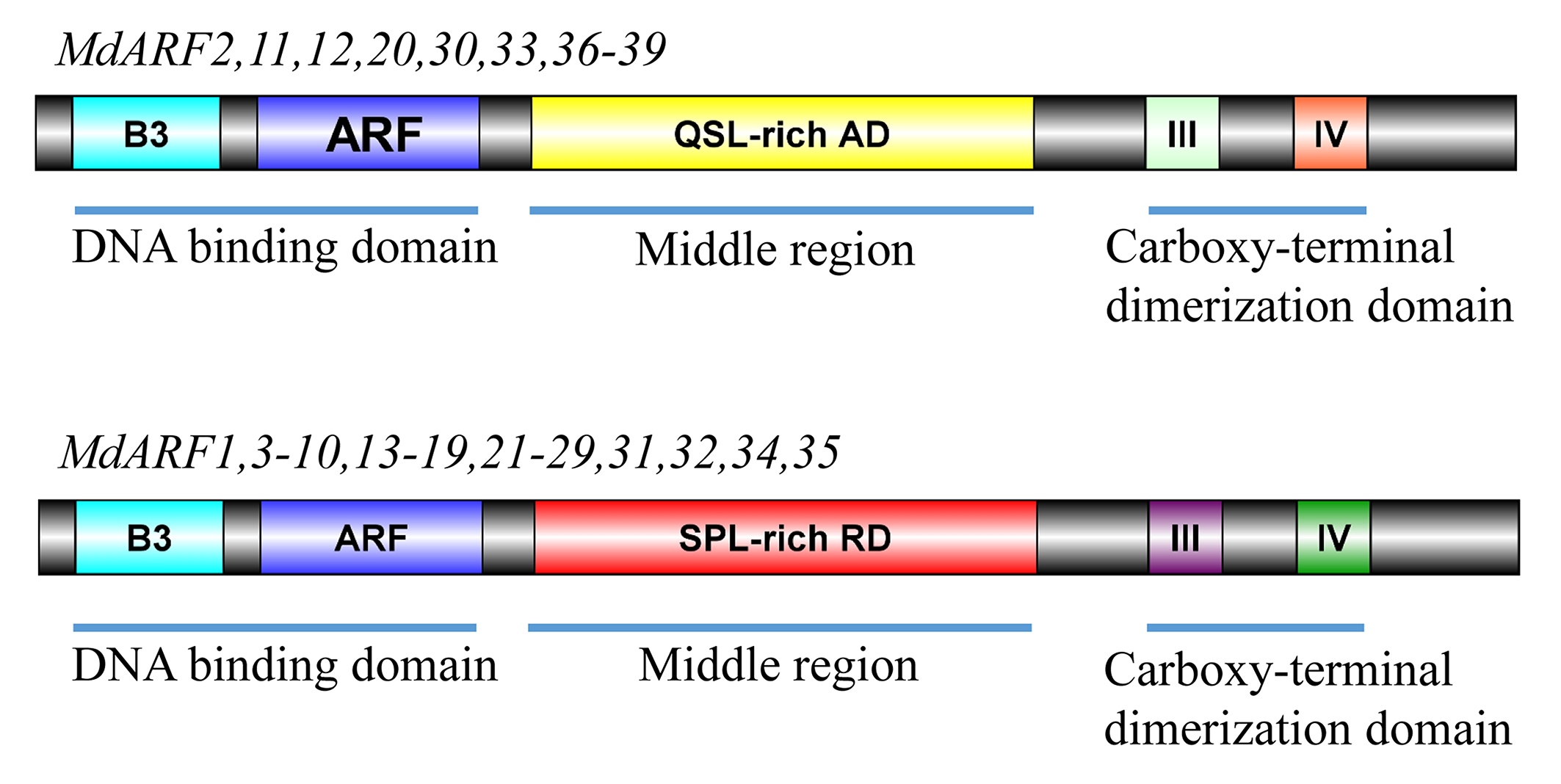

Supplement: Supplementary Figure 11 — The MxARF family of transcription factors in Malus domestic. MdARF2, 11, 12, 20, 30, 33, and 36–39 have an activation domain (AD) that is enriched in glutamine (Q), serine (S), and leucine (L). The remainder of the ARFs consist of transcriptional repressors with a repression domain (RD) that is enriched in serine (S) and in some cases proline (P). All ARFs contain a conserved DNA binding domain (DBD). [file Image11.TIF]

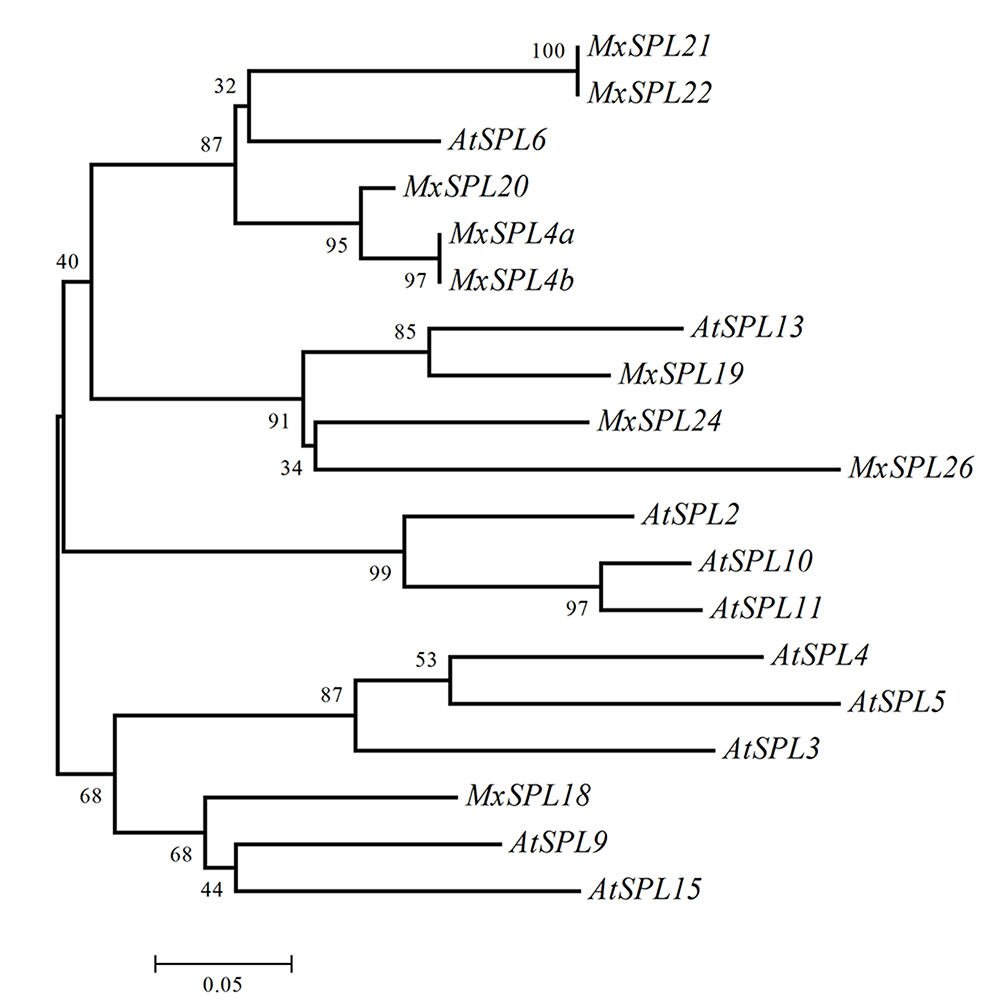

Supplement: Supplementary Figure 12 — Phylogenetic analysis of miR156-targeted SPL between apple and Arabidopsis. Phylogenetic tree was constructed with SBP domain protein sequences. Phylogenetic tree was constructed using MEGA 4.0 software with the neighbor-joining (NJ) method and the bootstrap test replicated 1,000 times. [file Image12.TIF]
