# Supplementary material for: High miR156 Expression Is Required for Auxin-Induced Adventitious Root Formation via MxSPL26 Independent of PINs and ARFs in Malus xiaojinensis
Source: Front Plant Sci. 2017 Jun 19;8:1059. doi: 10.3389/fpls.2017.01059 (PMC5474533; doi:10.3389/fpls.2017.01059)
Supplement: Supplementary Table 1 — Primers for constructing vector. [file Table1.DOCX]

**Supplementary Table 1.** Primers for constructing vector

| Primer name | DNA sequence (5’-3’) | Description |
| --- | --- | --- |
| *MdMIR156a*-F | GCTCTAGAGCTTTGCCGAACCAAGGAAT | Constructing miR156 overexpression vector |
| *MdMIR156a*-R | CAAGGATCCTCAAAAGTCACGCTCTCATT |  |
| *AtIPS1*-F | GTGGATCCAAGAAAAATGGCCATCCCCTAGC | Constructing mimicry156 expression vector |
| *AtIPS1*-R | TCCCCGGGCGAGGAATTCACTATAAAGAGAATCG |  |
| Mimicry156-F | ctTGACAGAAGATAGAAGTGAGCATtttctagagggagataa |  |
| Mimicry156-R | aaATGCTCACTTCTATCTTCTGTCAagcttcggttcccctcg |  |
| *MxSPL4a&4b-F* | GCTCTAGAATGGGTTGGGAACTGAAAATC | Constructing *miR156-resistant SPLs* overexpression vector |
| *MxSPL4a&4b-R* | CAAGGATCCTTACCTTATTTGCAGATCACT |  |
| *MxSPL18-F* | GCTCTAGAATGGGCTCGAGTTCTATGAC |  |
| *MxSPL18-R* | TCCCCCGGGTTAAAGTGACCAGTTCATCT |  |
| *MxSPL19-F* | GTGGATCCATGGACTGGAACTTGAAAGC |  |
| *MxSPL19-R* | TCCCCCGGGCTACTGCCAATGAAATGGAG |  |
| *MxSPL20-F* | GCTCTAGAATGGTGGGCAAACATTTGCA |  |
| *MxSPL20-R* | CAAGGATCCTTAAGTGATTCTGAGGCAGC |  |
| *MxSPL21&22-F* | GCTCTAGAATGGAGTCCTGGAGTTTTGG |  |
| *MxSPL21&22-R* | CAAGGATCCCTAAAGTTTCTCCCAGCCTC |  |
| *MxSPL24-F* | GCTCTAGAATGGAAGATGGATCAAAGGG |  |
| *MxSPL24-R* | TCCCCCGGGTCAGGAATAATCTGAATTCT |  |
| *MxSPL26-F* | GCTCTAGAATGGAGTGGGACTTCAAGGA |  |
| *MxSPL26-R* | CAAGGATCCTCAGTTGTTCGAAAAGCCAT |  |
| Mutant MxSPL4 -I | GCTGCGCATTAAGCTTGTTAAGTTCTCAATCACAGAAC |  |
| Mutant MxSPL4 -II | GAACTTAACAAGCTTAATGCGCAGCCAGAGTCTGAGAT |  |
| Mutant MxSPL18 -I | GCCGCGCATTAAGCTTGTTAAGTAATCAACCATGGGGC |  |
| Mutant MxSPL18 -II | TTACTTAACAAGCTTAATGCGCGGCTTGAGTCAGTGGC |  |
| Mutant MxSPL19 -I | ACTGCGCATTAAGCTTGTTAAGTTCACCGCAGACGCAG |  |
| Mutant MxSPL19 -II | GAACTTAACAAGCTTAATGCGCAGTCCGAATCGTGGGT |  |
| Mutant MxSPL20 -I | GCTGCGCACGAAGCTTGTTAAGTTCTCAATCACAGAAC |  |
| Mutant MxSPL20 -II | GAACTTAACAAGCTTCGTGCGCAGCCAGAGTTTGAGAT |  |
| Mutant MxSPL21&22 -I | GCTGCGCATTAAGCTTGTTAAGTGCTCAATCGCATAAC |  |
| Mutant MxSPL21&22 -II | GCACTTAACAAGCTTAATGCGCAGCTGGAGTTTGAAGC |  |
| Mutant MxSPL24 -I | ATCGCGCATTAAGCTTGTTAAGTTGGACACGAGCAACA |  |
| Mutant MxSPL24 -II | CAACTTAACAAGCTTAATGCGCGATTAGATTCTATGTC |  |
| Mutant MxSPL26 -I | TGGCTCGCCAAAAAGCACAAGGGGTTGCGCATTATACTTGTTAAGTt |  |
| Mutant MxSPL26 -II | TAACTGGATTTTGTGTTGGATGTGAACTTAACAAGTATAATGCGCAac |  |
