# Supplementary material for: High miR156 Expression Is Required for Auxin-Induced Adventitious Root Formation via MxSPL26 Independent of PINs and ARFs in Malus xiaojinensis
Source: Front Plant Sci. 2017 Jun 19;8:1059. doi: 10.3389/fpls.2017.01059 (PMC5474533; doi:10.3389/fpls.2017.01059)
Supplement: Supplementary Table 2 — PCR primers of genes in Malus xiaojinesis. [file Table2.DOCX]

**Supplementary Table 2.** PCR primers of genes in *Malus xiaojinesis*

| Primer name | DNA sequence (5’-3’) | Description |
| --- | --- | --- |
| miR156-F | tgcactagcgtgtgacagaaga | qRT-PCR analysis of miR156 expression |
| miR156-R | acatcgtatcgtgaag |  |
| miR156-RT | gtcacatcgtatcgtgaagctgcgcagctgatgtgacgtgctcac |  |
| 5s-F | tgcactagcgtgtagaggaacc |  |
| 5s-R | acatcgtatcgtgaag |  |
| 5s-RT | gtcacatcgtatcgtgaagctgcgcagctgatgtgactggattgg |  |
| *MxSPL4a&4b*-F | CCCATGTACCTTCATGGAAAGTCAAG | RT-PCR analysis of *MxSPLs* expression in *Malus xiaojinesis* |
| *MxSPL4a&4b*-R | GCAAATGTTTGCCCACCATTTCT |  |
| *MxSPL18*-F | AGGTTCAGCAGGCGGGACTA |  |
| *MxSPL18*-R | TCGTCGGAAAGTGATTGGAGG |  |
| *MxSPL19*-F | CCTCTGTTCAAAGCCGCAATCT |  |
| *MxSPL19*-R | ACTGGACATAAAACTCCCCGGAT |  |
| *MxSPL20*-F | GCCAAGAGATACTCCCTACTGACATTC |  |
| *MxSPL20*-R | CTGGAACTGCTGAAGCTGTGTTG |  |
| *MxSPL21&22*-F | CAAGATATTCGTGCGGATTCGATTAA |  |
| *MxSPL21&22*-R | CTGTGGAAATGTTGGAATTCTGATGC |  |
| *MxSPL24*-F | GCTTACCGAGAAAAACAAATCCCAT |  |
| *MxSPL24*-R | AGACTGAACTGGACTGTGCTGTGT |  |
| *MxSPL26*-F | GTCACCACTACTCATCCAGGTCAGA |  |
| *MxSPL26*-R | TCCTCCTCATCATGTGAGCATGAGAATT |  |
| *MxEF1α*-F | ATTCAAGTATGCCTGGGTGC |  |
| *MxEF1α*-R | CAGTCAGCCTGTGATGTTCC |  |
| *MxARF3_F* | ATGGCGAATCGGGAAGG | RT-PCR analysis of *MxARFs* expression in *Malus xiaojinesis* |
| *MxARF3_R* | CCCCTGTGGAAAGTAGAAGACTCTC |  |
| *MxARF4_F* | TGCTCGGGAATGAGGTTTAAG |  |
| *MxARF4_R* | GGCGAATTAGGCCAACAAATC |  |
| *MxARF7_F* | ATGCTGTGACTTTAACGAATTCCAG |  |
| *MxARF7_R* | GGCGAAGACTTAGATGGATACTGCA |  |
| *MxARF8_F* | AAACCTGATGATGTAAAGCCACCG |  |
| *MxARF8_R* | GAACTGGTGAGACTGTATCGCCA |  |
| *MxARF13_F* | TGGTGGTTTTGGTGACTATTCTGTT | RT-PCR analysis of *MxARFs* expression in *Malus xiaojinesis* |
| *MxARF13_R* | CCCGGATTCTCAAAAGGTGATG |  |
| *MxARF14_F* | CATGACCAGGTGGAAAAAGG |  |
| *MxARF14_R* | AAACACTGGCTCTATCTCTTGG |  |
| *MxARF15_F* | GCTAAACGATCCAAGAGGTTCAGG |  |
| *MxARF15_R* | CACAACCAACGAATTCCTCCC |  |
| *MxARF16_F* | ATGGCGTCTTCTACAGGTTTC |  |
| *MxARF16_R* | GAGATGGATGGATGGATGGTATG |  |
| *MxARF17_F* | GGTGATCTCTGTGTTGGGATTAG |  |
| *MxARF17_R* | AGCCAGAATTTCCAGCAGTAG |  |
| *MxARF8_F* | AAACCTGATGATGTAAAGCCACCG |  |
| *MxARF8_R* | GAACTGGTGAGACTGTATCGCCA |  |
| *MxARF19_F* | CTCCTACCAGAAGCAGATCAAA |  |
| *MxARF19_R* | GCAGTAAGTGTCTTGCAGAATG |  |
| *MxARF20_F* | CTTTCTCCTATAGCGACTCACAGCG |  |
| *MxARF20_R* | GGCAGCAAGAGAATTATTCCCAGTA |  |
| *MxARF21_F* | TCAACCGTGTGGCTTCTATC |  |
| *MxARF21_R* | TCCCATTGCTTGGGTTTCT |  |
| *MxARF23_F* | TGGGAATGGAGCATCAGACTTT |  |
| *MxARF23_R* | CGGATTCTGAACACCTAGACCG |  |
| *MxARF24_F* | GTAGCGGGGGATGCTGTGCTTTT |  |
| *MxARF24_R* | GCCTACAACTGAATCAGGAAGACCA |  |
| *MxARF25_F* | CAACCTACTGACTTTGACCTAGAA |  |
| *MxARF25_R* | CCATAAGAACTAAGAGCGGAGAG |  |
| *MxARF28_F* | GTTAGCCCATGGTTGGTAGAA |  |
| *MxARF28_R* | GTGTTGTGGTAGCCTCATCTT |  |
| *MxARF29_F* | GTTAGCCCATGGTTGGTAGAA |  |
| *MxARF29_R* | GTGTTGTGGTAGCCTCATCTT |  |
| *MxARF30_F* | GCATGTTCGGACTGGAAGGG |  |
| *MxARF30_R* | CAAGGATCATCCCCGACAAGTAG |  |
| *MxARF31_F* | TGGTAGATCAGAGAAGCAATGTTCC |  |
| *MxARF31_R* | AATCCTTTGCTAACCTCACCACG |  |
| *MxARF32_F* | CTCTGCCGGGTTATCAATGT |  |
| *MxARF32_R* | GCTCCTTCTCTACTGCATTCTC |  |
| *MxARF33_F* | TTAGTGGATCTACTGGAACAC |  |
| *MxARF33_R* | TGTTTCCTTCGGTGATAGTTGCAG |  |
| *MxARF34_F* | GGGATTGAAGGGCAACTAGAA |  |
| *MxARF34_R* | GATCATCGCCAACAAGAAGAATG |  |
| *MxARF35_F* | GTGAGCATAAGACACAGGAGAG |  |
| *MxARF35_R* | CCCAGTTACAGTCCCGTTATAG |  |
| *MxARF37_F* | GCTGCCCACACAGATTACTT |  |
| *MxARF37_R* | GAAAGGCAATGGTGCGTTATTC |  |
| *MxRTCS-like-F* | ACTCCAACAACAGGTCGCG | qRT-PCR analysis of *MxRTCS-like* expression in *Malus xiaojinesis* |
| *MxRTCS-like-R* | TGTGTGTCAACAGCATCAT |  |
| *MxPIN1_F* | CGTTTACGGGATGTCTGCG | qRT-PCR analysis of *MxPINs* expression in *Malus xiaojinesis* |
| *MxPIN1_R* | GCTTGCCCATTAGCCCTCTTT |  |
| *MxPIN3_F* | ACGCATCAAGGCGGTCACTC |  |
| *MxPIN3_R* | TACCGTCTGCGCGGGATAAA |  |
| *MxPIN4_F* | AAGCTCCATGTCACTGTCAGAAAAT |  |
| *MxPIN4_R* | CTCGGACTCGCATTGCTCAT |  |
| *MxPIN6_F* | CGAATCCGGCGCTCTACCT |  |
| *MxPIN6_R* | TGCTGTGAAAATCTCCCCGTC |  |
| *MxPIN8_F* | ACCATAAAGTTCACCATCACC |  |
| *MxPIN8_R* | TGTGACATTCTTGTTCTTCAAC |  |
| *MxPIN9_F* | CGTCTCCACTCTGCCCAACACTC |  |
| *MxPIN9_R* | CTGTCTTCCGTCGAGCGACATAAT |  |
| *MxPIN10_F* | CTGGACCAAAGTCAGCAAAAGGG |  |
| *MxPIN10_R* | AGCGACATAATATCGGAGTCGACGT |  |
| *MxPIN12_F* | CTAAGGAATACAATGTTCATCCAA |  |
| *MxPIN12_R* | GTTTGCTCGACATGATAAATAGA |  |
| *MxPIN13_F* | ATGTTGGTGTCCCTTCCG |  |
| *MxPIN13_R* | CTTTCCCCTCGATCGATC |  |
|  |  |  |
