# Supplementary material for: High miR156 Expression Is Required for Auxin-Induced Adventitious Root Formation via MxSPL26 Independent of PINs and ARFs in Malus xiaojinensis
Source: Front Plant Sci. 2017 Jun 19;8:1059. doi: 10.3389/fpls.2017.01059 (PMC5474533; doi:10.3389/fpls.2017.01059)
Supplement: Supplementary Table 3 — PCR primers of genes in Nicotiana benthamiana. [file Table3.DOCX]

**Supplementary Table 3.** PCR primers of genes in *Nicotiana benthamiana*

| Primer name | DNA sequence (5’-3’) | Description |
| --- | --- | --- |
| miR156-F | tgcactagcgtgtgacagaaga | qRT-PCR analysis of miR156 expression |
| miR156-R | acatcgtatcgtgaag |  |
| miR156-RT | gtcacatcgtatcgtgaagctgcgcagctgatgtgacgtgctcac |  |
| 5s-F | tgcactagcgtgtagaggaacc |  |
| 5s-R | acatcgtatcgtgaag |  |
| 5s-RT | gtcacatcgtatcgtgaagctgcgcagctgatgtgactggattgg |  |
| *NbSPL5a*-F | GACAGACACTCATCAGAT | qRT-PCR analysis of *NbSPLs* expression in transgenic tobacco lines |
| *NbSPL5a*-R | GAGCAAGCATATCGTATC |  |
| *NbSPL5b*-F | CATCTTTCGTATCCCTTTCTG |  |
| *NbSPL5b*-R | TTCCTTACAAGCCGTGAA |  |
| *NbSPL2a*-F | CAGTGTAGCAGGTTCCATAGTT |  |
| *NbSPL2a*-R | AGACTGAACCAGTTGGGAATG |  |
| *NbSPL2b*-F | GCAGTGTAGCAGGTTCCATAG |  |
| *NbSPL2b*-R | GACTGAACCAGTTGGGAATGA |  |
| *NbSPL15a*-F | TGGCTTCTGCTTCTTCTTCTAC |  |
| *NbSPL15a*-R | CCCTCTTCCTTTCTTGGACATC |  |
| *NbSPL15b*-F | GTTCAGTCACCAGGACCAATTA |  |
| *NbSPL15b*-R | GCTTGAGAAGTGATTCGTAGGT |  |
| *NbEF1α*-F | CACGCATTGCTTGCTTTCA |  |
| *NbEF1α*-R | TCCATCTTGTTACAGCAGCAAATC |  |
| *NbARF1-F* | CATTGCAGCATTCACAGAAGACG | RT-PCR analysis of *NbARFs* expression in transgenic tobacco lines |
| *NbARF1-R* | GCAGGGTAAAGCGGGGTGGTATT |  |
| *NbARF2-F* | GGAGAGCCATAACCAAAGCGAC |  |
| *NbARF2-R* | AGGCTAGCTGATGTAATCTGAGACTG |  |
| *NbARF4-F* | CAAGGGCGGTAGTAAATGGTGA |  |
| *NbARF4-R* | GGTTTGTAAGTGCGAAGAAGC |  |
| *NbARF5-F* | ATCAGCCTAAGTGTTGCCGTTGT |  |
| *NbARF5-R* | TTTACTTGCTCGGACAGTTCACC |  |
| *NbARF6-F* | AATACCGACTCTTTGAACATACTGC |  |
| *NbARF6-R* | GTTCTATTAGTTGGGTTTGCTTGG |  |
| *NbARF7-F* | CTGGAGTTCTGTAAGATGGTCAGGAGG |  |
| *NbARF7-R* | GAAAAAGCACGTCTATTCGTTAAGCC |  |
| *NbARF8-F* | TCAGGGCTTCTCCTCCCGACA |  |
| *NbARF8-R* | TCCAGTGACCTCCCAACAGACC |  |
| *NbARF9-F* | ATCGTACGAGGCGTTACACAAG |  |
| *NbARF9-R* | TGCTGCTTGCATCCGTTAAA |  |
| *NbARF10-F* | TGTTTGTGTTTCCTTACGTTTCG |  |
| *NbARF10-R* | GCATGCTCAGTGTGGATTATCG |  |
| *NbARF11-F* | GAGGGGACACTACGATGACAACT |  |
| *NbARF11-R* | ACATGCACGGACTGCCAGAT |  |
| *NbARF12-F* | AGATGCTAACAAATGCTTCCCTC | RT-PCR analysis of *NbARFs* expression in transgenic tobacco lines |
| *NbARF12-R* | TGTAGTTTGCCAGTTTCATCCCT |  |
| *NbARF16-F* | CAAGCCGTCTAACACCAACT |  |
| *NbARF16-R* | ACCAGCAGACTCCAACTCAAGAAT |  |
| *NbARF18-F* | AACAGACATGATGCCAGAG |  |
| *NbARF18-R* | GAGTTCGTCAACAGCGTAT |  |
| *NbARF19-F* | CAAGGACAAGCCCTACCGAG |  |
| *NbARF19-R* | TCCCACCGAACCTTCAGAC |  |
| *NbARF22-F* | TACCCGCCCATAGCCTATAA |  |
| *NbARF22-R* | AATGTCTCCATCCCTCCATTTC |  |
| *NbARF23-F* | CTGTGCGAGCTAGGAGTAGAGGG |  |
| *NbARF23-R* | TGGGATTAGGAGGGAAGAAGATG |  |
| *NbPIN1-F* | GGAGCTGCAGCACAACAAAGT | RT-PCR analysis of *NbPINs* expression in transgenic tobacco lines |
| *NbPIN1-R* | ACCTTTCTTGTTATTAGTGC |  |
| *NbPIN1b-F* | GGTGCAAAAGCAGTGGGTT |  |
| *NbPIN1b-R* | CTTACATCCTTAGCTGTAT |  |
| *NbPIN3-F* | GCAACAAGTACATTTACAG |  |
| *NbPIN3-R* | ATCAGTGCCACCAAACAC |  |
| *NbPIN3b-F* | GTGCTCAGGGAGCTCTAAC |  |
| *NbPIN3b-R* | ATCATGGCTAGCCCTACC |  |
| *NbPIN4-F* | GCAGTCCCTTTACTTTCC |  |
| *NbPIN4-R* | CCATTCTAGGCTACCATTT |  |
| *NbPIN7* | CAACCCTTCCAAACACCTTAATC |  |
| *NbPIN7* | CTCGAGCTCAACTGCTACTTC |  |
| *NbPIN9-F* | AATCACATGGTGGTCTTA |  |
| *NbPIN9-R* | ATAAACCCCATTTCCTCTCCC |  |
| *NbRTCS-like-F* | AGCGGCTGTTACAATCTCTTAT | qRT-PCR analysis of *NbRTCS-like* expression in transgenic tobacco lines |
| *NbRTCS-like-R* | GGGTTTGTATAGTTGGAACCATTT |  |
